# An Efficient Solvent-Free Synthesis of 2-Hydroxy-2-(trifluoromethyl)-2*H*-chromenes Using Silica-Immobilized L-Proline

**DOI:** 10.3390/molecules181011964

**Published:** 2013-09-26

**Authors:** Cuilian Xu, Guoyu Yang, Caixia Wang, Sufang Fan, Lixia Xie, Ya Gao

**Affiliations:** School of Science, Henan Agricultural University, Zhengzhou 450002, China; E-Mails: ygy1096@sina.com.cn (G.Y.); wcx670815@163.com (C.W.); fansufang123@163.com (S.F.); toxielix@163.com (L.X.); 15136423631@163.com (Y.G.)

**Keywords:** solvent-free, 2-hydroxy-2-(trifluoromethyl)-2*H*-chromene-3-carboxylates, synthesis, Knoevenagel condensation, silica-immobilized L-proline

## Abstract

An efficient synthesis of 2-hydroxy-2-(trifluoromethyl)-2*H*-chromene-3-carboxylates was carried out under solvent-free conditions in an oven or microwave oven via the Knoevenagel condensation of salicylaldehydes with ethyl trifluoroacetoacetate followed by intramolecular cyclization in the presence of silica-immobilized L-proline. The structures of the title compounds were characterized by IR, ^1^H-NMR, ^13^C-NMR, HRMS and X-ray single crystal diffraction. The improved method described herein is economical, easily-operated and environmentally friendly. Furthermore, the catalyst can be recovered conveniently and reused without obvious loss of activity.

## 1. Introduction

The chromene ring moiety has been identified as one of the privileged scaffolds for drug discovery due to its broad spectrum of biological activity [1,2,3]. Among various chromene isomers, 2*H*-chromenes are attracting much more interest from chemists because compounds that possess this group show a variety of activities, including antiviral [4,5], anti-tumor [6,7], anti-bacterial/antimicrobial [8,9], fungicidal [10], antioxidative [11], insecticidal agents [12], and activator of potassium channels effects [13,14].

On the other hand, although introduction of fluorine atoms into organic compounds has been known as one of the best strategies for the enhancement or modification of their original biological activities [15,16,17,18,19], up to now there are limited reports on the preparation of 2-fluoroalkylated 2*H*-chromenes. Laurent *et al.* reported the synthesis of 2-(trifluoromethyl)- and 2-(perfluoroalkyl)-2-hydroxy-2*H*-chromenes by intramolecular cyclization of 3-(perfluoroalkyl)-3-phenoxypropenals in the presence of aluminum chloride [20]. Wang *et al*. reported the synthesis of 2-trifluoromethyl-2*H*-benzopyran-3-carboxylic acids from the reaction of substituted salicylaldehydes with ethyl trifluorocrotonate and found these potential as novel potent and selective cyclooxygenase-2 inhibitors [21,22,23]. Recently Chizhov *et al.* prepared ethyl 6-substituted 2-hydroxy-2-(trifluoromethyl)-2*H*-chromene-3-carboxylates via the Knoevenagel condensation of salicylaldehydes with ethyl trifluoroacetoacetate in the presence of piperidinium acetate [24]. Li synthesized novel coumarin dyes bearing trifluoromethyl substituents by the condensation of 3-(trifluoroacetyl)coumarins with various arylhydrazines and studied their fluorescence activities [25]. Zhu’s group reported a Lewis acid-promoted reaction of 2-(trifluoromethyl)-2-hydroxy-2*H*-chromenes with indoles and thiophenols, and obtained both 2-functionalized-2-trifluoromethyl-3-ethoxycarbonyl-2*H*-chromenes and 4-functionalized-2-trifluoro-methyl-3-ethoxycarbonyl-4*H*-chromenes [26,27]. To the best of our knowledge, however, there are no reports on the synthesis of 2-hydroxy-2-(trifluoromethyl)-2*H*-chromenes under solvent-free conditions.

Recently the progress in the field of solvent-free reactions has provided organic chemists with an efficient synthetic method of great promise [28,29,30]. Particularly this technique has been coupled with microwave-assisted organic synthesis (MAO), resulting in clean, easy-to-perform, cheap, safe and environmentally friendly conditions which are widely used as synthetic tools under “Green Chemistry” conditions [31,32]. Difficult recycling of homogeneous catalysts, such as piperidinium acetate, prompted us to find a suitable heterogeneous catalyst. L-Proline and its analogues have been extensively investigated as catalysts for many reactions; much effort has been dedicated to the immobilization and recycling of L-proline and its analogues with the assistance of organic and inorganic supports [33,34,35].

In continuation of our work on green synthetic strategies for the preparation of heterocyclic compounds [36,37], we were prompted to use a solvent-free methodology for the synthesis of 2-hydroxy-2-(trifluoromethyl)-2*H*-chromenes from salicylaldehydes and ethyl trifluoroacetoacetate under solvent-free conditions in the presence of silica-immobilized L-proline (L-proline/SiO_2_).

## 2. Results and Discussion

### 2.1. Synthesis

**Scheme 1:**
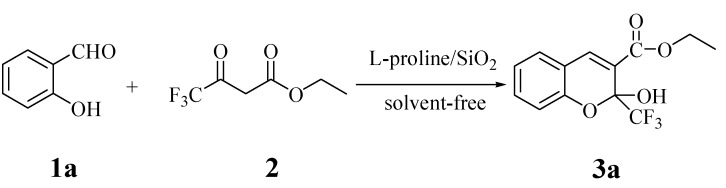
Solvent-free synthesis of compound **3a**.

Initially, the reaction n of salicylaldehyde (**1a**) and ethyl trifluoroacetoacetate (**2**) was tested without any catalyst under neat conditions, but almost no product was obtained without or with microwave irradiation (Scheme 1 and Table 1, entries 1–2). After adding 20 mol% of L-proline/SiO_2_, the reaction afforded the product **3a** in 80% (entry 5) and 69% (entry 3) yield, respectively, with or without MW irradiation (MWI), under solvent-free conditions. Thus, the reaction could be catalyzed by L-proline/SiO_2_ and the reaction rate could be increased by MWI. Increasing the loading of catalyst improved the yield and shortened the reaction time (entries 4–7), however, the yield did not increase when the amount of the catalyst was more than 30 mol% of substrate **1a** (entry 7). Next, the reaction with different ratios of **1a** to **2** was examined under 30 mol% L-proline/SiO_2_ catalysis. It was observed that the variation of this ratio had a great influence on the yield. The yield of **3a** reached a maximum at the molar ratio of 1:1.5 and 1:2. When the quantity of compound **2** continued to increase, yield was reduced and more side-products were observed according to HPLC (entries 10–11). Therefore, the ratio of **1a** to **2** was optimized as 1:1.5. After the product was extracted thoroughly with dichloromethane, the separated catalyst was subjected to another cycle with fresh reactants under similar conditions. It was observed that the yield was nearly the same. The above procedure was repeated for three cycles, and no substantial loss in the catalytic activity of the immobilized catalyst was observed (entries 12–13).

**Table 1:** Optimization for the synthesis of 2*H*-chromene **3a** ^a^.

Entry	Mol ratio of 1a and 2	Loading of catalyst (mol%) ^a^	Time	Yield ^b^ (%)
1	1:1.5	0	6 h ^c^	0.5
2	1:1.5	0	20 min	0.7
3	1:1.5	20	6 h ^c^	69
4	1:1.5	10	15 min	38
5	1:1.5	20	18 min	80
6	1:1.5	30	14 min	82
7	1:1.5	40	9 min	82
8	1:1	30	17 min	43
9	1:2	30	14 min	82
10	1:2.5	30	15 min	56
11	1:3	30	15 min	38
12	1:1.5	30	14 min	82 ^d^
13	1:1.5	30	14 min	80 ^e^

^a^ Reaction conditions: **1a** (7 mmol), L-proline/SiO_2_ (4.5 g), MWI (126 W). ^b^ Isolated yield based on **1**. All yields are the average of two runs based on fresh catalyst. ^c^ Heating in the oven at 80 °C under solvent-free condition. ^d^ Catalyst was reused in the second run. ^e^ Catalyst was reused in the third run.

**Scheme 2:**
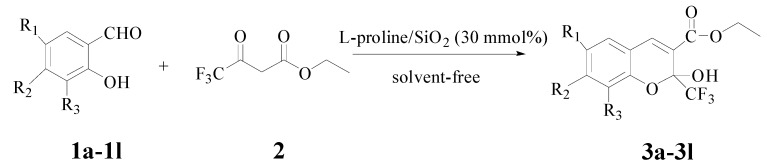
Solvent-free synthesis of compound **3a**–**l**.

To evaluate the efficiency of this methodology, various substituted salicylaldehydes **1b**–**1l** were next reacted with ethyl trifluoroacetoacetate under optimal conditions (Scheme 2). The results are shown in Table 2.

**Table 2:** Synthesis of 2-hydroxy-2-(trifluoromethyl)-2*H*-chromenes **3a**–**l** under optimum conditions.

Entry	R_1_	R_2_	R_3_	Product	MWI (126 W)		Without MWI ^b^	
					Time (min)	Yield ^a^ (%)	Time (h)	Yield ^a^ (%)
1	H	H	H	**3a**	12	82	6	75
2	Cl	H	H	**3b**	17	89	6	81
3	Br	H	H	**3c**	16	85	6	77
4	Cl	H	Cl	**3d**	12	87	8	80
5	Br	H	Br	**3e**	16	88	6	81
6	H	H	OMe	**3f**	12	70	6	69
7	H	OMe	H	**3g**	8	68	8	65
8	H	H	OEt	**3h**	16	72	8	66
9	Me	H	H	**3i**	18	81	6	65
10	H	OH	H	**3j**	17	66	8	67
11	NO_2_	H	H	**3k**	6	92	2	83
12	H	-CH=CH-CH=CH-		**3l**	18	74	8	68

^a^ Isolated yield based on **1**. ^b^ Heating in the oven at 80 °C under solvent-free conditions.

As can be seen from Table 2, electron-withdrawing (entries 2–5, entry 11) and electron-donating groups (entries 7–10, entry 12) at various positions of the benzene rings are well tolerated. The aromatic aldehydes with electron-donating groups afforded lower yields in comparision with those with electron-withdrawing groups. For instance, 4-methoxy-2-hydroxylbenzaldehyde (**1g**) and 4-hydroxy-2-hydroxylbenzaldehyde (**1j**) gave products **3g** and **3j** with yields of 68% and 66% under MWI, respectively (entries 7 and 10), but compounds **1b** and **1k** afforded the products **3b** and **3k** with yields of 89% and 92% (entries 2 and 11), respectively. On the other hand, the reactions required longer times and gave relatively lower yields by the alternative method employing heating in the oven at 80 °C. In most cases the microwave-assisted conditions were found to be superior to those without MWI, and the chromenes **3a**–**3l** were obtained in better yields (66%–92%), compared with yields of 65%–83% in the same reaction without MWI.

**Scheme 3:**
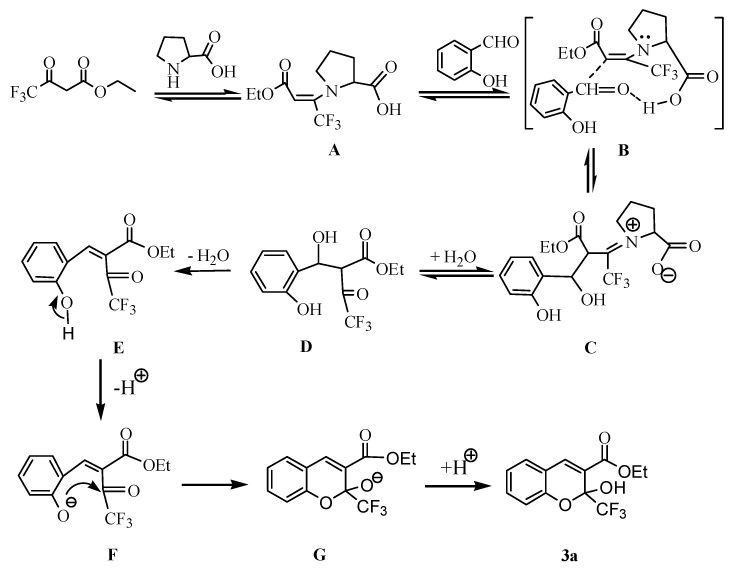
Proposed mechanism.

The proposed catalytic cycle is shown in Scheme 3. The L-proline-catalyzed reaction proceeds via an enamine intermediate **A**. Intermediate **A** reacts with salicylaldehyde via transition state **B** to give intermediate **C**, which produced the Knoevenagel product **E** through hydrolysis and dehydration. The subsequent cyclization occurs to yield **3a** by addition of phenoxide ion to the more electrophilic carbonyl group rather than to the ester group forming intermediate **G**.

### 2.2. Structural Characterization of Chromenes ***3a**–**l***

The chemical structures of chromenes **3a**–**l** were characterized by IR, ^1^H-NMR, ^13^C-NMR and HRMS. All of the data in the spectra were in good accordance with the structures. The IR spectra of **3a**–**l** displayed OH absorption in the range 3110–3443 cm^−1^, and the intensive absorption bands in the range 1671–1721 cm^−1^ attributed to the C=Os in the ester groups. The diagnostic signal for the proton H-4 in chromenes **3a**–**l** appeared at 7.64–8.47 ppm, which is usually in lower field than common aromatic protons are. The signal for -OH at C-2 in **3a**–**l**, which appeared at 7.22–9.60 ppm, was shifted downfield because of the formation of intromolecular H-bonding between the OH and O atom in the carboxyl group and neighboring electron-withdrawing CF_3_ group. In the ^13^C-NMR spectra of **3a**–**l**, the quartets of CF_3_ and C-2 atom with their corresponding coupling constants ^1^*J*_C,F_ = 289–291 Hz and ^2^*J*_C,F_ = 33.6–36.4 Hz, appeared at 122.0–123.0 ppm and 95.2–96.6 ppm respectively, similar to the related data [24,38]. Compounds **3a**–**l** all showed the molecular-ion peak [M+Na]^+^ in the high resolution mass spectrum, matching with the caculated data.

**Figure 1:**
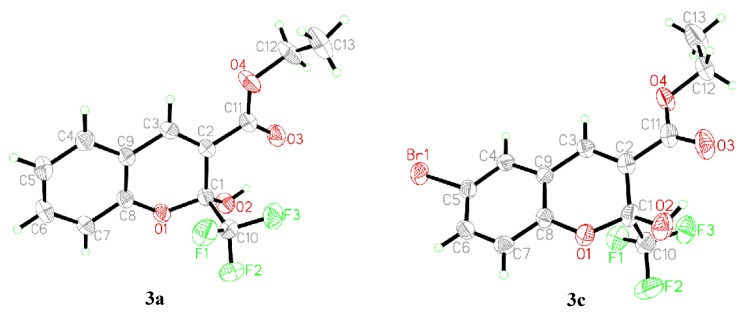
The molecular structures of **3a** and **3c**, showing the atomic numbering scheme and displacement ellipsoids draw at the 30% probability level.

**Figure 2:**
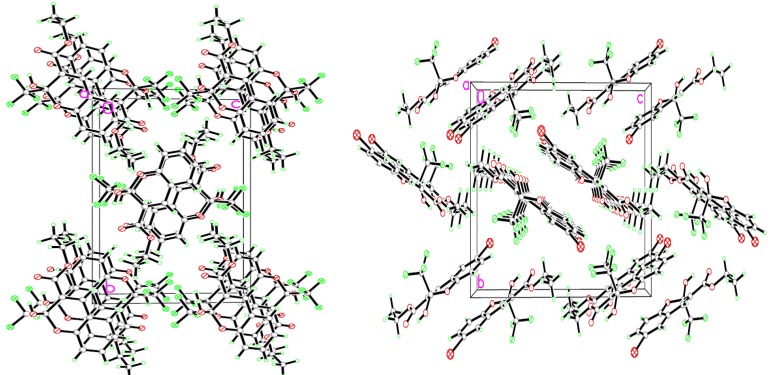
View of the molecular packing in **3a** and **3c**.

The structures of 2*H*-chromenes were further confirmed by the X-ray diffraction determination of single crystals of compounds **3a** and **3c** (single crystal X-ray diffraction data of compounds **3a** and **3c** are deposited with CCDC Nos. 845964 and 845962, respectively). The perspective and packing views are shown in Figure 1a,b and Figure 2a,b respectively. The crystal data and refinement details are given in Table 3. It is seen that compounds **3a** and **3c** are isomorphous, and they crystallize in the monoclinic space P21/c with four molecules in the unit cell. The value of O1-C8 bond length is 1.374 (5) Å in **3c**, which is slightly shorter, compared with 1.396(4) Å in **3a**. This is probably due to the inductive negative effect of the halogen atom on the lactone O atom (O1) lone pair of electrons. In **3a** and **3c** molecules, the carboxylate carbonyl groups (O3) are out of the plane defined by atoms C2-C9 by 3.6 and 6.6°, respectively. The 2-hydroxy groups (O2) are out of the plane defined by atoms O1-C9 by 2.5 and 3.4°, respectively. The above mentioned hydroxyl deviations from planarity seem to attribute to sp^3^ hybridization of C1. It is interesting to note that the replacement of H by Br does not alter the space group. In the crystal structures of chromenes **3a** and **3c**, no intermolecular hydrogen-bonds are formed because compounds **3a** and **3c** present an anti conformation between the ethoxy (O4) and the hydroxy (O2). Thereby, the carboxylate carbonyl O atom (O3) acts as a hydrogen-bond acceptor allowing the formation of intramolecular hydrogen-bond, and the detailed data for intromolecular hydrogen bond are shown in Table 4. Molecules of **3a** and **3c** are packed in an offset face-to-face arrangement and form a layered stack.

**Table 3:** Crystal data and structure refinement for **3a** and **3c**.

	3a	3c
formula	C_13_H_11_F_3_O_4_	C_13_H_10_BrF_3_O_4_
*Fw*	288.22	367.12
crystal system	monoclinic	monoclinic
space group	*P2(1)/c*	*P2(1)/c*
*a* (Å)	8.415 (1)	7.085 (2)
*b* (Å)	16.604 (3)	12.831(3)
*c* (Å)	9.443 (9)	15.526 (3)
*β* (deg)	97.51 (3)	93.52 (3)
*V* (Å^3^)	1308.2 (5)	1408.9 (5)
*Z*	4	4
*T* (K)	293 (2)	293 (2)
*D*_calc_ (Mg m^−3^)	1.463	1.731
*μ* (mm^−1^)	0.135	2.964
*R_1_ (I>2σ(I))*	0.0792	0.0492
*R_1_* (all data)	0.0829	0.0651
*wR_2_ (I>2σ(I))*	0.2261	0.1433
*wR_2_* (all data)	0.2310	0.1553
*GOOF*	1.052	1.074

**Table 4:** Hydrogen bonding distances [Å] and angles [°] for **3a** and **3c**.

Crystal	D-H· A	d(D-H)	d(D·A)	D-H· A
**3a**	O2-H2· O3	0.82	2.71 (1)	143
**3c**	O2-H2· O3	0.82	2.64 (1)	147

## 3. Experimental

### 3.1. Gereral

Infrared spectra were recorded with a Nicolet IS10 Fourier Transform Infrared Spectrophotometer (4,000–400 cm^−1^) (KBr pellets). ^1^H and ^13^C-NMR spectra of CDCl_3_ solutions were obtained on a Bruker DPX-400 or Advance 300 Spectrometer, respectively. ^19^F-NMR spectra were recorded in CDCl_3_ without an internal standard. HPLC analyses for the qualitative and quantitative analysis of the products were carried out using an Agilent 1200 pump equipped with an Agilent 1200 detector. High resolution mass spectrometry data were measured on a Waters Q-Tof micro^TM^ instructment with an electrospray ionization source (ESIMS). X-ray diffraction data were collected on a Rigaku RAXIAS-IV type diffractometer. Melting points were determined on a X-5 digital microscopic melting-point apparatus (Beijing Tech Instruments Co., Beijing, China) and are uncorrected. A household microwave oven (Haier MM-2270MG, Qingdao, China) and electrothermal drying oven (Qin Stewart 101-2AB, Tianjin, China) were used for heating the reaction mixtures.

X-ray Crystallography parameters for data collection and refinement of the compounds are summarized in Table 1. Intensities were collected on a Rigaku Saturn 724 CCD diffractometer (Mo-Kα, λ = 0.71073 Å) at a temperature of 293 K using the SMART and SAINT programs [39]. The structures were solved by direct method and refined on F2 by full-matrix least-squares methods with SHELXTL-97 crystallographic software package [40]. All the non-hydrogen atoms were refined with anisotropic thermal displacement coefficients. The hydrogen atoms were assigned with common isotropic displacement factors and included in the final refinement by using geometrical restrains.

All solvents and reagents were used without futher purification.

### 3.2. Preparation of Catalyst L-Proline/SiO_2_

Silica (45 g, 200 mesh) was added to a solution of L-proline (22 mmol) in deionized water (50 mL). After being stirred at room temperature for 30 min, the mixture was first dried at room temperature overnight, and then heated in an oven for 6 h at 50 °C. The resulting immobilized catalyst was kept in a desiccator for use.

### 3.3. General Synthetic Procedures for Compounds ***3a**–**3l***

#### 3.3.1. Oven Heating Procedure

In a typical experiment of Knoevenagel condensation reaction catalyzed by immobillized L-proline, aldehyde (7 mmol), ethyl trifluoroacetoacetate (14 mmol) and 4.5 g of L-proline/SiO_2_ were thoroughly ground in a mortar. The mixture was charged in a microwave tube (capacity 10 mL), then sealed with polytetrafluoroethylene film and heated in an oven at 80 °C for 6–8 h (monitored by HPLC). The mixture was allowed to cool to room temperature. Ethyl acetate was added and the resulting mixture was filtered, and the residue was sequentially washed with ethyl acetate or dichloromethane for at least three times. The combined solution was evaporated under reduced pressure, and the crude product was recrystallized from ethanol or ethyl acetate.

#### 3.3.2. Microwave Irradiation Procedure

The same procedure and dosage was applied as above. After being mixed fully, the mixture was put into a microwave tube, sealed, irradiated in the microwave oven under 126 W power. The reaction was monitored by HPLC. After the aldehyde had consumed, the irradiation was terminated and the mixture was allowed to cool to room temperature. The same work-up was as above.

*Ethyl 2-hydroxy-2-(trifluoromethyl)-2H-chromene-3-carboxylate* (**3a**) [24]. White solid, yield 76%, m.p. 103.4-104.2 °C (Lit. 102.0-103.0 °C). IR cm^−1^: 3,300, 1,695, 1,636, 1,608, 1,575, 1,489; ^1^H-NMR (CDCl_3_, 300 MHz) δ: 1.39 (t, *J* = 7.2 Hz, 3H, CH_3_), 4.36 (q, *J* = 7.2 Hz, 2H, CH_2_), 6.99-7.03 (m, 2H, H-6, H-8), 7.25 (dd, *J* = 7.9, 1.7 Hz, 1H, H-5), 7.36 (m, 1H, H-7), 7.54 (s, 1H, OH), 7.78 (s, 1H, H-4); ^13^C-NMR (CDCl_3_, 75 MHz) δ: 13.9 (CH_3_), 62.6 (CH_2_), 95.2 (q, ^2^*J*_C,F_ = 34.8 Hz, *C*CF_3_), 114.6, 115.8, 117.5, 122.6 (q, ^1^*J*_C,F_ = 290 Hz, CF_3_), 122.6, 129.5, 133.9, 139.3, 152.5, 166.7 (C=O); HRMS: calcd for *m/z* (C_13_H_11_F_3_O_4_ + Na)^+^: 311.0507; found: 311.0536.

*Ethyl 6-chloro-2-hydroxy-2-(trifluoromethyl)-2H-chromene-3-carboxylate* (**3b**). White solid, m.p. 115.5–116.9 °C. IR cm^−1^: 3,314, 1,700, 1,636, 1,565, 1,460; ^1^H-NMR (CDCl_3_, 300 MHz) δ: 1.40 (t, *J* = 7.2 Hz, 3H, CH_3_), 4.37 (q, *J* = 7.2 Hz, 2H, CH_2_), 6.98 (d, *J* = 8.7 Hz, 1H, H-8), 7.24 (d, *J* = 2.5 Hz, H-5), 7.31 (dd,* J* = 8.7, 2.5 Hz, H-7), 7.50 (s, 1H, OH), 7.70 (s, 1H, H-4); ^13^C NMR (CDCl_3_, 75 MHz) δ: 13.9 (CH_3_), 62.7 (OCH_2_), 95.4 (q, ^2^*J*_C,F_ = 35.1 Hz, *C*CF_3_), 116.0, 117.4, 118.7, 122.2 (q, ^1^*J*_C,F_ = 291 Hz, CF_3_), 127.6, 128.6, 133.4, 137.9, 151.0, 166.4 (C=O); HRMS: calcd for *m/z* (C_13_H_10_ClF_3_O_4_ + Na)^+^: 345.0118; found: 345.0135.

*Ethyl 6-bromo-2-hydroxy-2-(trifluoromethyl)-2H-chromene-3-carboxylate* (**3c**) [24]. Light yellow solid, m.p. 107.0–108.4 °C. IR cm^−1^: 3,248, 1,684, 1,629, 1,600, 1,564, 1,478; ^1^H-NMR (CDCl_3_, 300 MHz) δ: 1.39 (t, *J* = 7.2 Hz, 3H, CH_3_), 4.40 (q, *J* = 7.2 Hz, 2H, CH_2_), 6.90 (d, *J* = 8.7 Hz, 1H, H-8), 7.38 (d, *J* = 2.4 Hz, H-5), 7.43 (dd,* J* = 8.7, 2.4 Hz, H-7), 7.47 (s, 1H, OH), 7.70 (s, 1H, H-4); ^13^C-NMR (CDCl_3_, 75 MHz) δ: 13.9 (CH_3_), 62.7 (OCH_2_), 95.3 (q, ^2^*J*_C,F_ = 35.0 Hz, *C*CF_3_), 114.6, 115.9, 117.7, 119.1, 122.4 (q, ^1^*J*_C,F_ = 290 Hz, CF_3_), 131.6, 136.3, 137.8, 151.4, 166.3 (C=O); HRMS: calcd for *m/z* (C_13_ H_10_BrF_3_O_4_ + Na)^+^: 388.9613; found: 388.9634.

*Ethyl 6,8-dichloro-2-hydroxy-2-(trifluoromethyl)-2H-chromene-3-carboxylate* (**3d**). White solid, m.p. 129.6–131.3 °C. IR cm^−1^: 3,443, 1,709, 1,631, 1,564, 1,457; ^1^H-NMR (CDCl_3_, 300 MHz) δ: 1.41 (t, *J* = 7.2 Hz, 3H, CH_3_), 4.41 (q, *J* = 7.2 Hz, 2H, CH_2_), 7.17 (d, *J* = 2.4 Hz, 1H, H-7), 7.41 (d, *J* = 2.4 Hz, 1H, H-5), 7.56 (broad, 1H, OH), 7.69 (s, 1H, H-4); ^19^F-NMR (CDCl_3_, 376.5 MHz) δ: -87.15 (s, 3F); ^13^C-NMR (CDCl_3_, 75 MHz) δ: 13.9 (CH_3_), 62.9 (OCH_2_), 96.0 (q, ^2^*J*_C,F_ = 35.3 Hz, *C*CF_3_), 117.0, 119.7, 122.1, 122.2 (q, ^1^*J*_C,F_ = 290 Hz, CF_3_), 127.1, 127.5, 133.3, 137.3, 147.0, 166.0 (C=O); HRMS: calcd for *m/z* (C_13_H_9_Cl_2_F_3_O_4_ + Na)^+^: 378.9728; found: 378.9755.

*Ethyl 6,8-dibromo-2-hydroxy-2-(trifluoromethyl)-2H-chromene-3-carboxylate* (**3e**). White solid, m.p. 114.0–115.1 °C. IR cm^−1^: 3,251, 1,678, 1,624, 1,554, 1,470; ^1^H-NMR (CDCl_3_, 300 MHz) δ: 1.39 (t, *J* = 5.4 Hz, 3H, CH_3_), 4.38 (q, *J* = 5.4 Hz, 2H, CH_2_), 7.33 (d, *J* = 2.4 Hz, 1H, H-7), 7.47 (s, 1H, OH), 7.64 (s, 1H, H-4), 7.70 (d, *J* = 2.4 Hz, 1H, H-5); ^13^C-NMR (CDCl_3_, 100 MHz) δ: 14.0 (CH_3_), 62.9 (CH_2_), 95.6 (q, ^2^*J*_C,F_ = 36.4 Hz, *C*CF_3_), 110.9, 114.7, 116.9, 120.1, 122.2 (q, ^1^*J*_C,F_ = 290 Hz, CF_3_), 130.7, 137.3, 138.8, 148.6, 166.0 (C=O); HRMS: calcd for *m/z* (C_13_H_9_Br_2_F_3_O_4_ + Na)^+^: 466.8700; found: 466.8764.

*Ethyl 2-hydroxy-8-methoxy-2-(trifluoromethyl)-2H-chromene-3-carboxylate* (**3f**). Light yellow solid, m.p. 99.4–99.9 °C. IR cm^−1^: 3,328, 1,699, 1,637, 1,610, 1,581, 1,483; ^1^H-NMR (CDCl_3_, 300 MHz) δ: 1.40 (t, *J* = 7.2 Hz, 3H, CH_3_), 3.89 (s, 3H, CH_3_O), 4.37 (q, *J* = 7.2 Hz, 2H, CH_2_), 6.86 (dd, *J* = 7.2, 1.9 Hz, 1H, H-7), 6.96–7.02 (m, 2H, H-5, H-6), 7.50 (s, 1H, OH), 7.76 (s, 1H, H-4); ^13^C-NMR (CDCl_3_, 75 MHz) δ: 14.0 (CH_3_), 56.4 (OCH_3_), 62.4 (CH_2_), 95.4 (q, ^2^*J*_C,F_ = 34.8 Hz, *C*CF_3_), 114.8, 117.0, 118.2, 121.2, 122.4, 122.6 (q, ^1^*J*_C,F_ = 290 Hz, CF_3_), 139.4, 142.0, 147.5, 166.7 (C=O); HRMS: calcd for *m/z* (C_14_H_13_F_3_O_5_ + Na)^+^: 341.0623; found: 341.0692.

*Ethyl 2-hydroxy-7-methoxy-2-(trifluoromethyl)-2H-chromene-3-carboxylate* (**3g**). White solid, m.p. 76.0–76.5 °C. IR cm^−1^: 3,326, 1,684, 1,629, 1,569, 1,512, 1,469; ^1^H-NMR (CDCl_3_, 400 MHz) δ: 1.38 (t, *J* = 7.2 Hz, 3H, CH_3_), 3.82 (s, 3H, CH_3_O), 4.34 (q, *J* = 7.2 Hz, 2H, CH_2_), 6.56–6.58 (m, 2H, H-5, H-8), 7.16 (dd, *J* = 7.2, 2.0 Hz, 1H, H-6), 7.60 (s, 1H, OH), 7.74 (s, 1H, H-4); ^13^C-NMR (CDCl_3_, 100 MHz) δ: 14.0 (CH_3_), 55.6 (CH_3_O), 62.1 (CH_2_), 95.6 (q, ^2^*J*_C,F_ = 34.8 Hz, *C*CF_3_), 101.0, 109.7, 110.9, 111.2, 122.8 (q, ^1^*J*_C,F_ = 291 Hz, CF_3_), 130.7, 139.4, 154.5, 164.6, 167.1 (C=O); HRMS: calcd for *m/z* (C_14_H_13_F_3_O_5_ + Na)^+^: 341.0623; found: 341.0664.

*Ethyl* *8-ethoxy-2-hydroxy-2-(trifluoromethyl)-2H-chromene-3-carboxylate* (**3h**). White solid, m.p. 112.5–113.9 °C. IR cm^−1^: 3,301, 1,681, 1,632, 1,610, 1577, 1,486; ^1^H-NMR (CDCl_3_, 300 MHz) δ: 1.37–1.44 (m, 6H, 2CH_3_), 4.07–4.17 (m, 2H, CH_2_), 4.37 (q, *J* = 7.2 Hz, 2H, CH_2_), 6.85–6.88 (dd, *J* = 7.5, 1.7 Hz, 1H, H-7), 6.91–7.03 (m, 2H, H-5, H-6), 7.46 (s, 1H, OH), 7.76 (s, 1H, H-4); ^1^^3^C-NMR (CDCl_3_, 75 MHz) δ: 13.9 (CH_3_), 14.9 (CH_3_), 62.3 (CH_2_), 65.4 (CH_2_), 95.9 (q, ^2^*J*_C,F_ = 34.8 Hz, *C*CF_3_), 114.7, 118.3, 119.3, 121.4, 122.3, 122.6 (q, ^1^*J*_C,F_ = 290 Hz, CF_3_), 139.6, 142.5, 146.7, 166.7 (C=O); HRMS: calcd for *m/z* (C_15_H_15_F_3_O_5_ + Na)^+^: 355.0769; found: 355.0752.

*Ethyl 2-hydroxy-6-methyl-2-(trifluoromethyl)-2H-chromene-3-carboxylate* (**3i**). White solid, m.p. 81.8–82.9 °C. IR cm^−1^: 3,315, 1,682, 1,633, 1,613, 1,582, 1,493; ^1^H-NMR (CDCl_3_, 400 MHz) δ: 1.37 (t, *J* = 7.2 Hz, 3H, CH_3_), 2.28 (s, 3H, CH_3_), 4.36 (q, *J* = 7.2 Hz, 2H, CH_2_), 6.90 (d,* J* = 8.4 Hz, 1H, H-8), 7.04 (s, 1H, H-5), 7.11 (dd, *J* = 8.4, 1.6 Hz, 1H, H-7), 7.22 (s, 1H, OH), 7.73 (s, 1H, H-4); ^13^C-NMR (CDCl_3_, 100 MHz) δ: 13.9 (CH_3_), 20.2 (CH_3_), 62.3 (CH_2_), 95.3 (q, ^2^*J*_C,F_ = 34.7 Hz, *C*CF_3_), 114.6, 115.6, 117.3, 122.7 (q, ^1^*J*_C,F_ = 291 Hz, CF_3_), 129.6, 132.1, 134.6, 139.5, 150.5, 166.8 (C=O); HRMS: calcd for *m/z* (C_14_H_13_F_3_O_4_ + Na)^+^: 325.0664; found: 325.0666.

*Ethyl 2,7-dihydroxy-2-(trifluoromethyl)-2H-chromene-3-carboxylate* (**3j**). White solid, m.p. 152.0–153.2 °C. IR cm^−1^: 3,110, 1,696, 1,608, 1,510, 1,460; ^1^H-NMR (CDCl_3_, 400 MHz) δ: 1.26 (t, *J* = 7.2 Hz, 3H, CH_3_), 4.14–4.25 (m, 2H, CH_2_), 6.38 (s, 1H, H-8), 6.49 (dd, *J* = 8.4, 1.6 Hz, 1H, H-6), 7.34 (d, *J* = 8.4 Hz, 1H, H-5), 7.87 (s, 1H, H-4), 8.83 (broad, 1H, OH), 10.37 (broad, 1H, OH); ^13^C-NMR (CDCl_3_, 100 MHz) δ: 14.3 (CH_3_), 60.5 (CH_2_), 96.3 (q, ^2^*J*_C,F_ = 40.0 Hz, *C*CF_3_), 102.1, 109.6, 110.5, 114.8, 122.6 (q, ^1^*J*_C,F_ = 288 Hz, CF_3_), 131.3, 139.4, 154.0, 162.7, 163.4 (C=O); HRMS: calcd for *m/z* (C_14_H_11_F_3_O_5_ + Na)^+^: 327.0456; found: 327.0455.

*Ethyl 2-hydroxy-6-nitro-2-(trifluoromethyl)-2H-chromene-3-carboxylate* (**3k**) [24]. Light yellow solid, m.p. 120.0–120.7 °C (Lit. 120–120.5 °C). IR cm^−1^: 3,426, 1,721, 1,619, 1,517, 1,478; ^1^H-NMR (CDCl_3_, 400 MHz) δ: 1.32 (t, *J* = 7.2 Hz, 3H, CH_3_), 4.29 (q, *J* = 7.2 Hz, 2H, CH_2_), 7.28 (d, *J* = 9.2 Hz, 1H, H-8), 8.19 (s, 1H, H-4), 8.29 (dd, *J* = 9.2, 2.4 Hz, 1H, H-7), 8.61 (d, *J* = 2.4 Hz, 1H, H-5), 9.60 (broad, 1H, OH); ^13^C-NMR (CDCl_3_, 100 MHz) δ: 14.1 (CH_3_), 61.3 (CH_2_), 97.1 (q, ^2^*J*_C,F_ = 34.5 Hz, *C*CF_3_), 116.8, 117.7, 121.5, 122.1 (q, ^1^*J*_C,F_ = 288 Hz, CF_3_), 125.6, 128.5, 137.0, 142.4, 156.5, 162.6 (C=O). HRMS: calcd for *m/z* (C_1__3_H_1__0_F_3_O_6_ + Na)^+^: 356.0358; found: 356.0360.

*Ethyl 2-hydroxy-2-(trifluoromethyl)-2H-benzo[h]chromene-3-carboxylate* (**3l**). Yellow solid, m.p. 123.9.0–124.8 °C. IR cm^−1^: 3,224, 1,671, 1,614, 1,592, 1,570, 1,519, 1,465; ^1^H-NMR (CDCl_3_, 400 MHz) δ: 1.48 (t, *J* = 7.2 Hz, 3H, CH_3_), 4.46 (q, *J* = 7.2 Hz, 2H, CH_2_), 7.24 (m, 1H, ArH), 7.48 (m, 1H, ArH), 7.62 (m, 1H, ArH), 7.80–7.82 (m, 2H, ArH, OH), 7.89 (m, 1H, ArH), 8.06 (m, 1H, ArH), 8.47 (d, *J* = 3.6 Hz, 1H, ArH); ^13^C-NMR (CDCl_3_, 100 MHz) δ: 14.1 (CH_3_), 62.5 (CH_2_), 95.6 (q, ^2^*J*_C,F_ = 35.0 Hz, *C*CF_3_), 110.6, 112.3, 116.6, 120.8, 122.7 (q, ^1^*J*_C,F_ = 291 Hz, CF_3_), 125.0, 128.3, 128.9, 129.5, 130.1, 134.9, 135.1, 152.2, 167.0 (C=O). HRMS: calcd for *m/z* (C_17_H_13_F_3_O_4_ + Na)^+^: 361.0664; found: 361.0663.

## 4. Conclusion

In summary, silica-immobilized L-proline has been employed as an efficient catalyst for the solvent-free preparation of 2-hydroxy-2-(trifluoromethyl)-2*H*-chromene-3-carboxylates. The reaction proceeded via a tandem condensation-cyclization process and gave the title products in good yields. This environmentally friendly synthetic method possesses such advantages as operational simplicity, environmentally friendliness, good catalytic performance, reusability, and reduction of time when combined with MW irradiation.

## Supplementary Materials

Supplementary materials can be accessed at: http://www.mdpi.com/1420-3049/18/10/11964/s1.
