# Supplementary material for: An Efficient Solvent-Free Synthesis of 2-Hydroxy-2-(trifluoromethyl)-2H-chromenes Using Silica-Immobilized L-Proline
Source: Molecules. 2013 Sep 26;18(10):11964–77. doi: 10.3390/molecules181011964 (PMC6290574; doi:10.3390/molecules181011964)

Supplementary Materials

IR spectra of compounds **3a–l**
^{1}H-NMR and ^{13}C-NMR of compounds **3a–l**
^{19}F-NMR of compound **3d**
HRMS of compounds **3a–l**

IR spectra of compounds 3a–l

IR spectra of compounds 3a

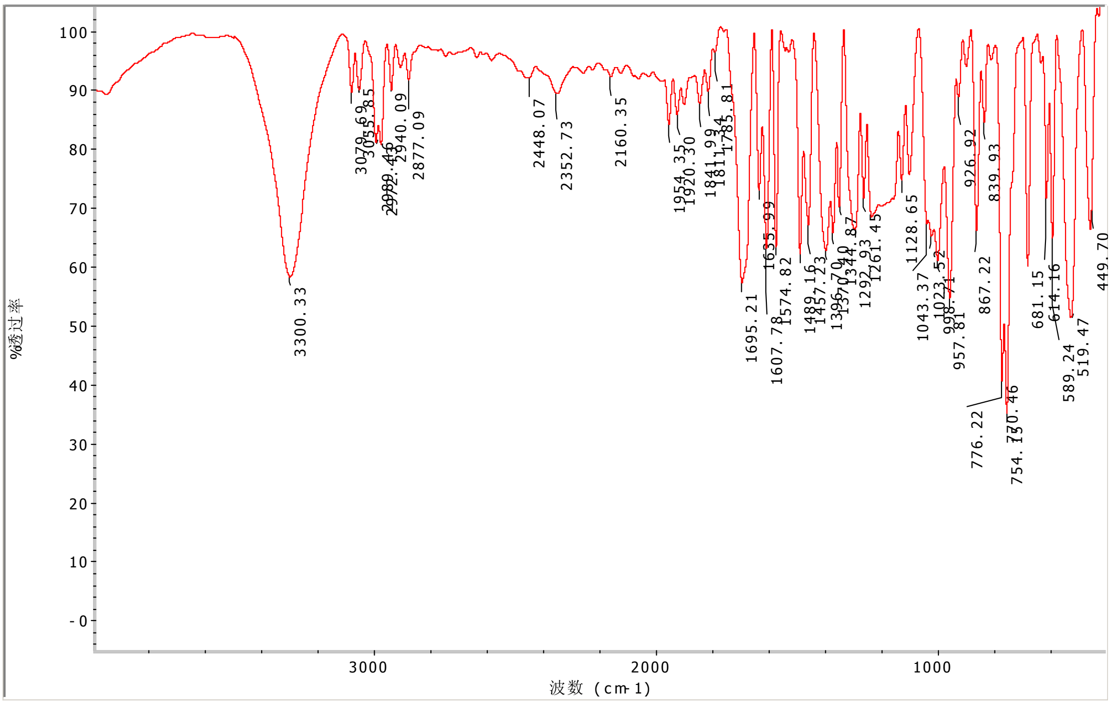

IR spectra of compounds 3b

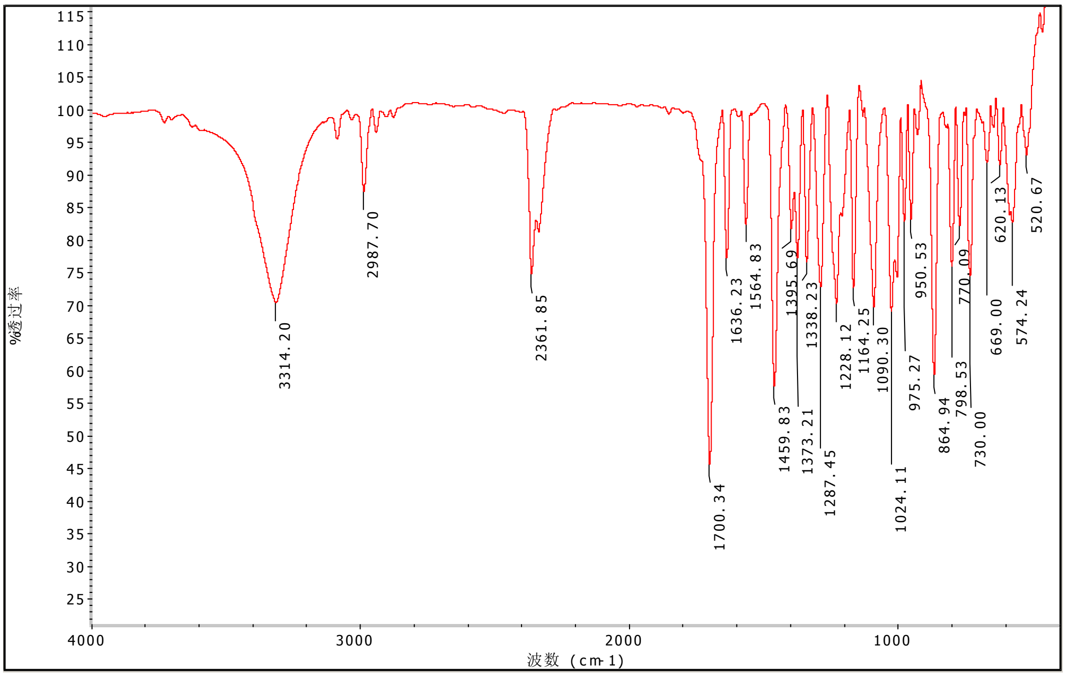

IR spectra of compounds **3c**

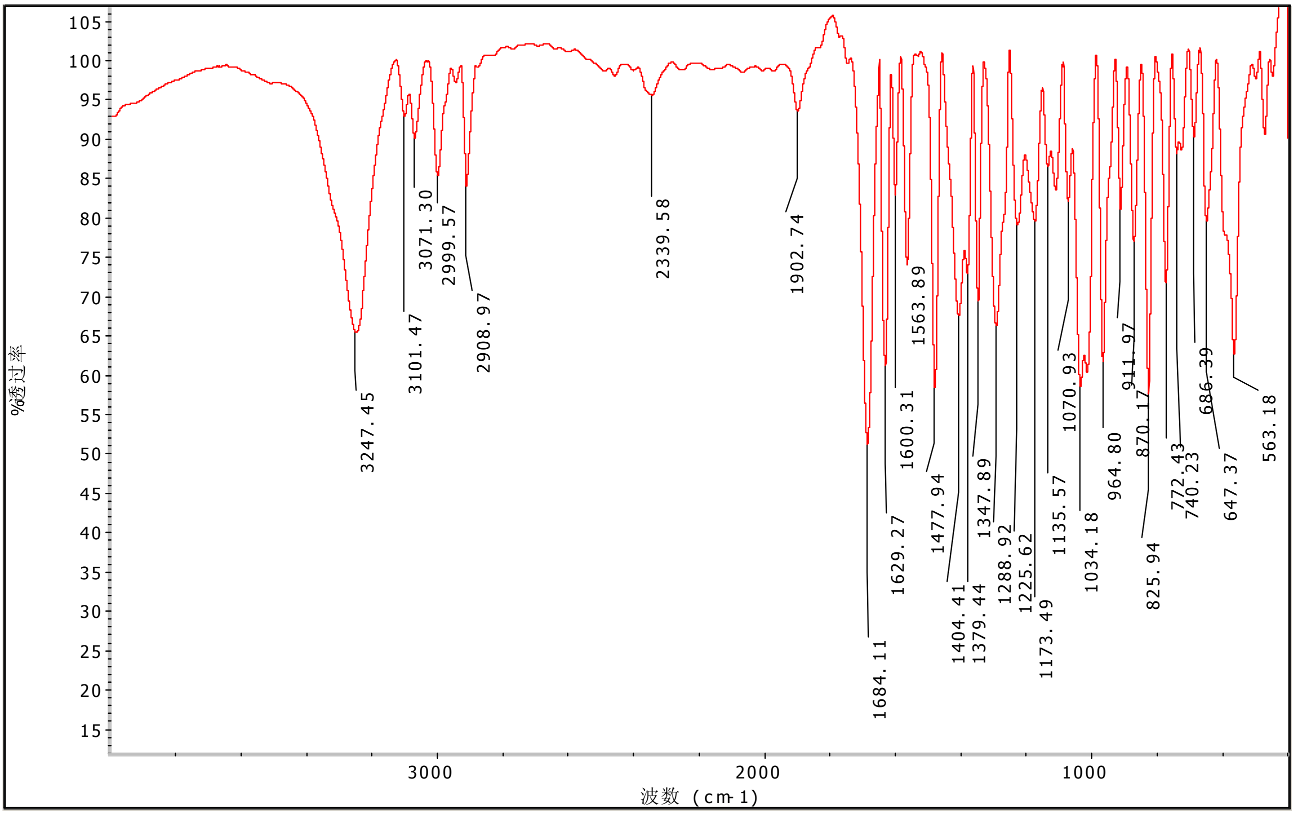

IR spectra of compounds **3d**

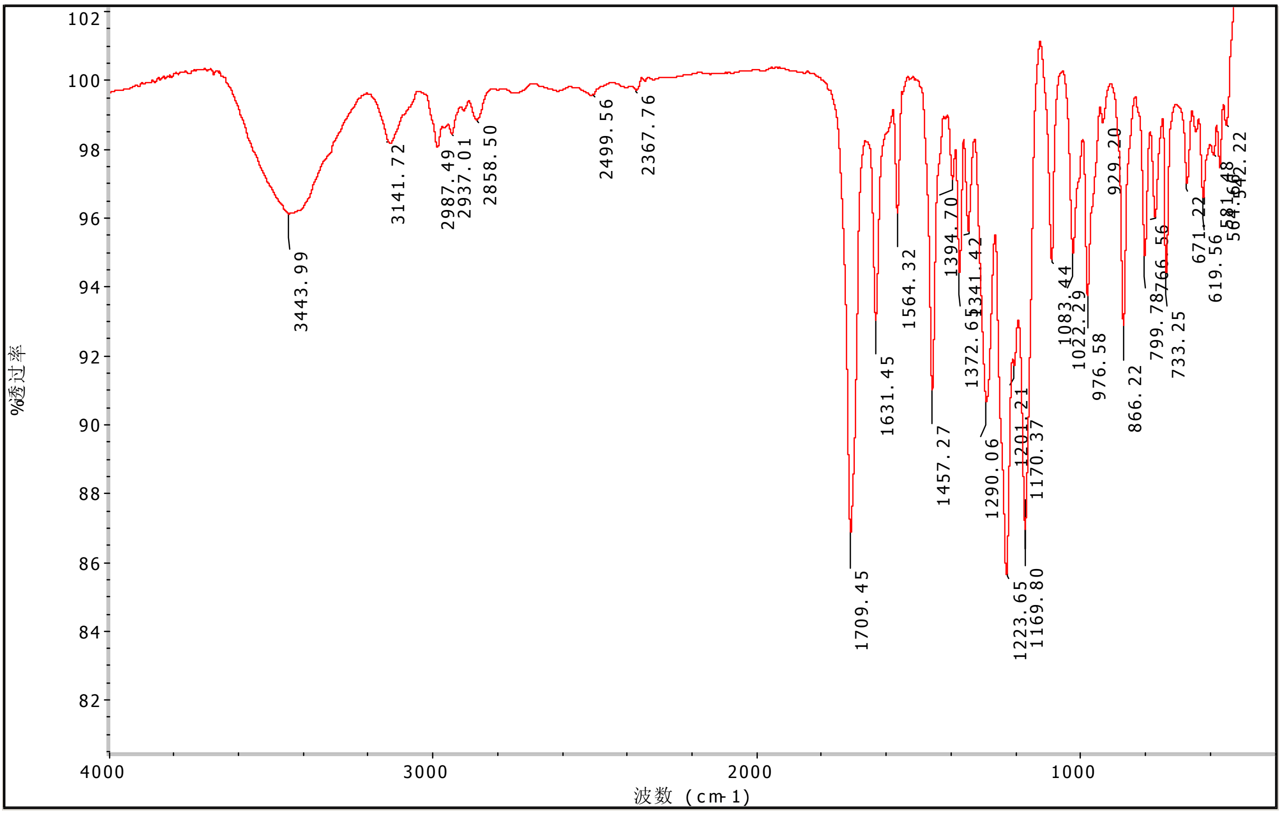

IR spectra of compounds **3e**

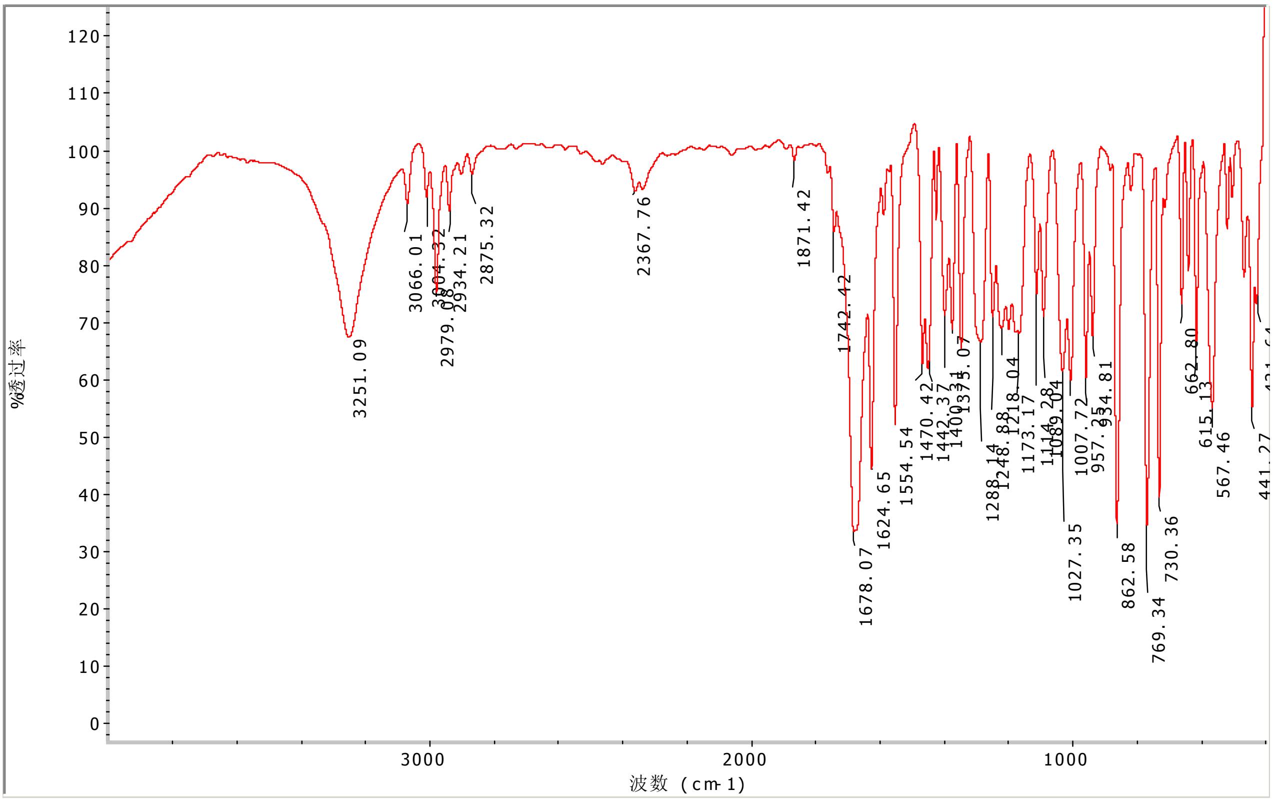

IR spectra of compounds **3f**

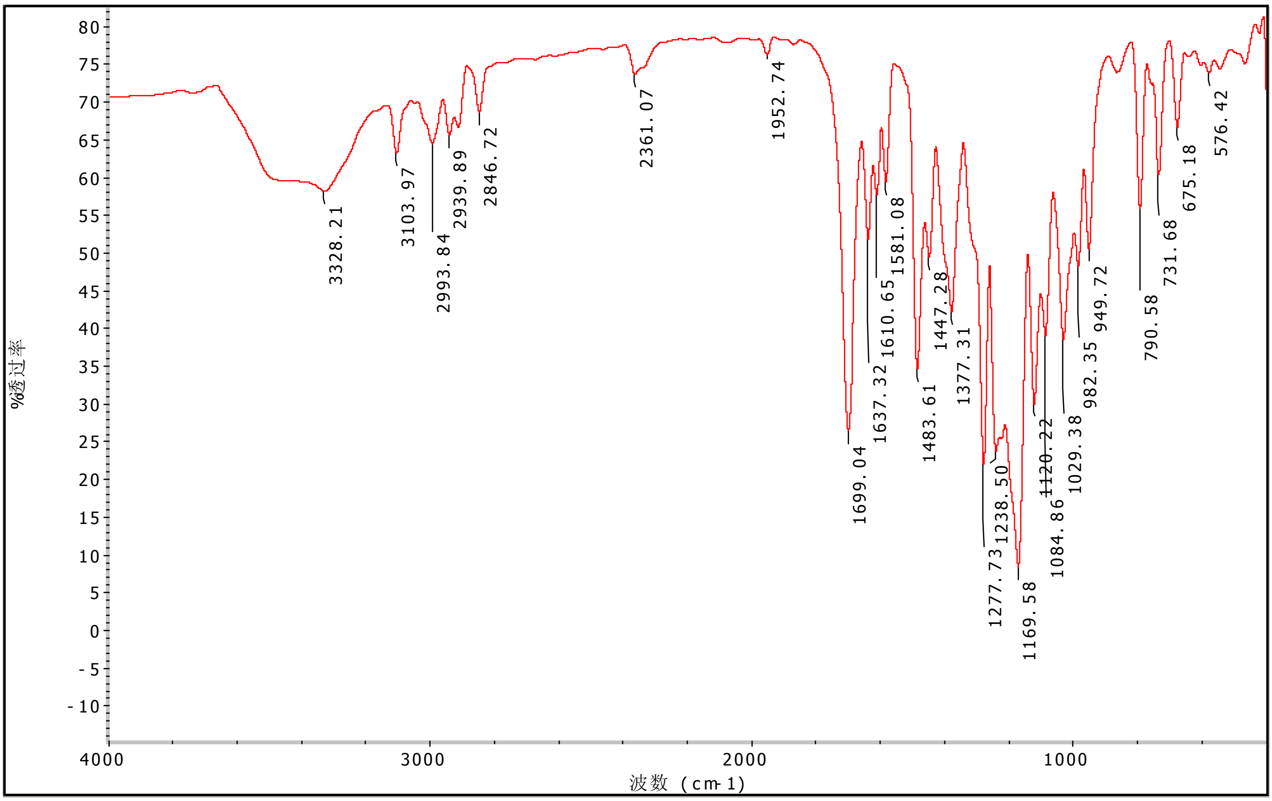

IR spectra of compounds **3g**

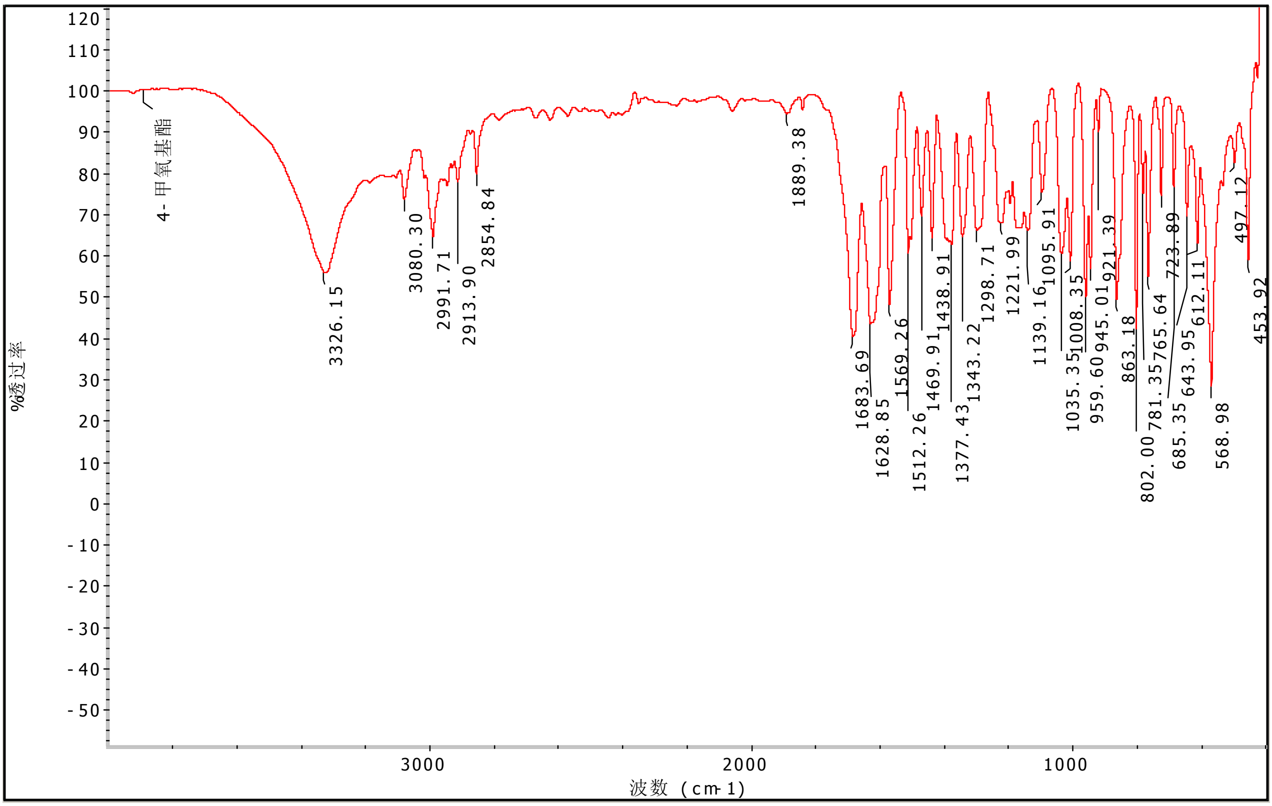

IR spectra of compounds **3h**

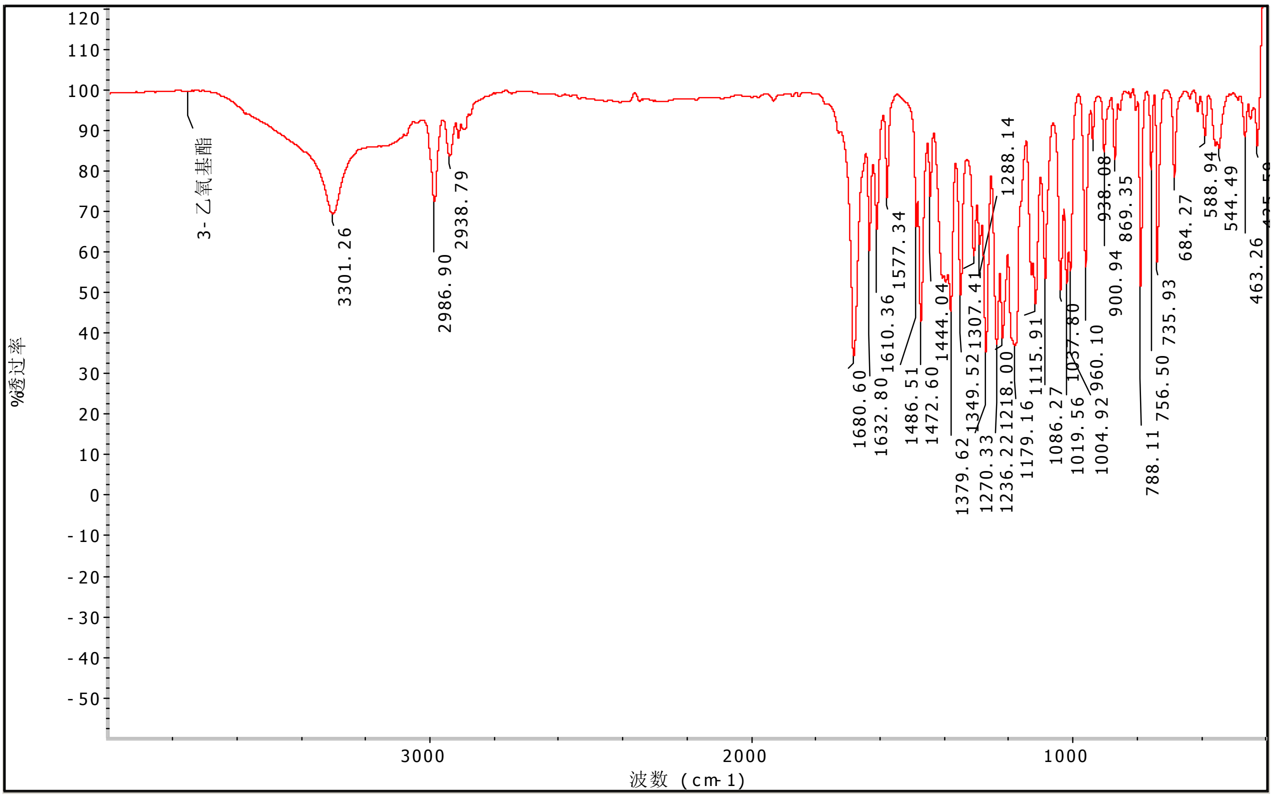

IR spectra of compounds **3i**

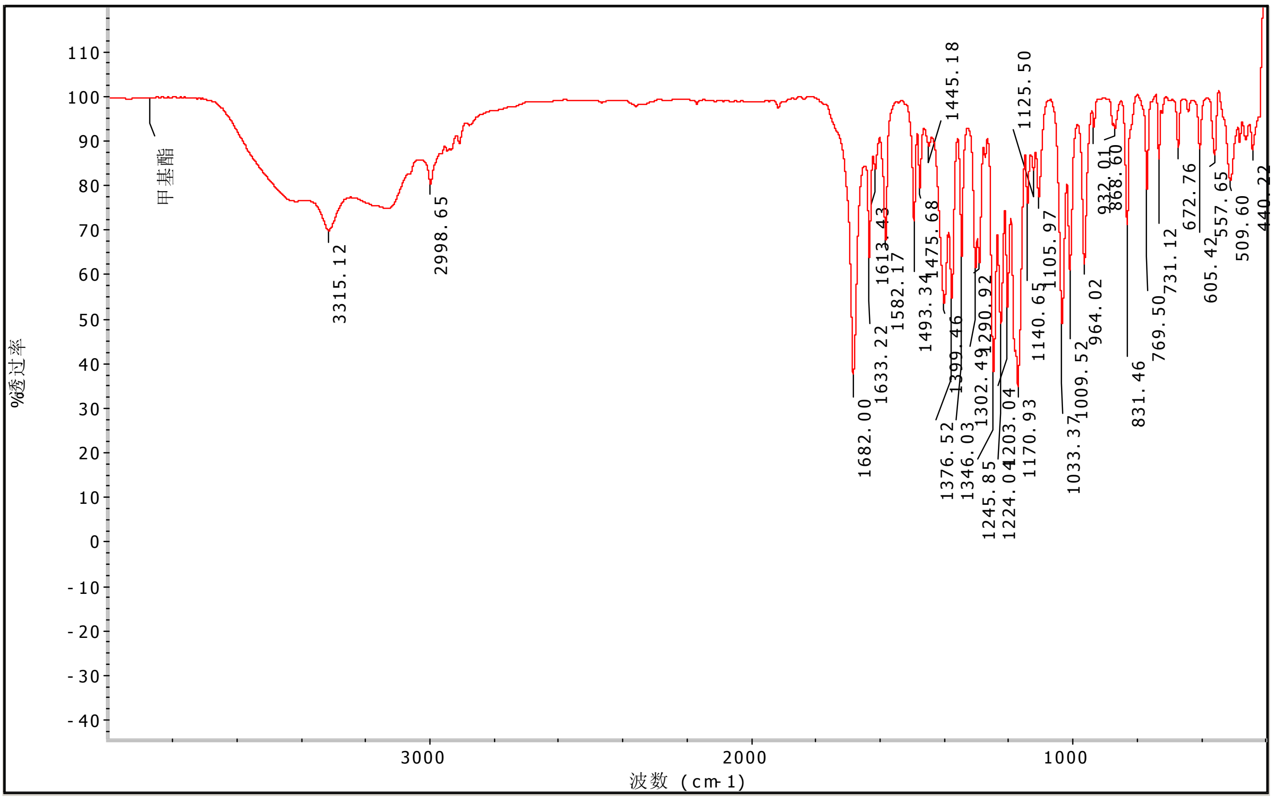

IR spectra of compounds **3j**

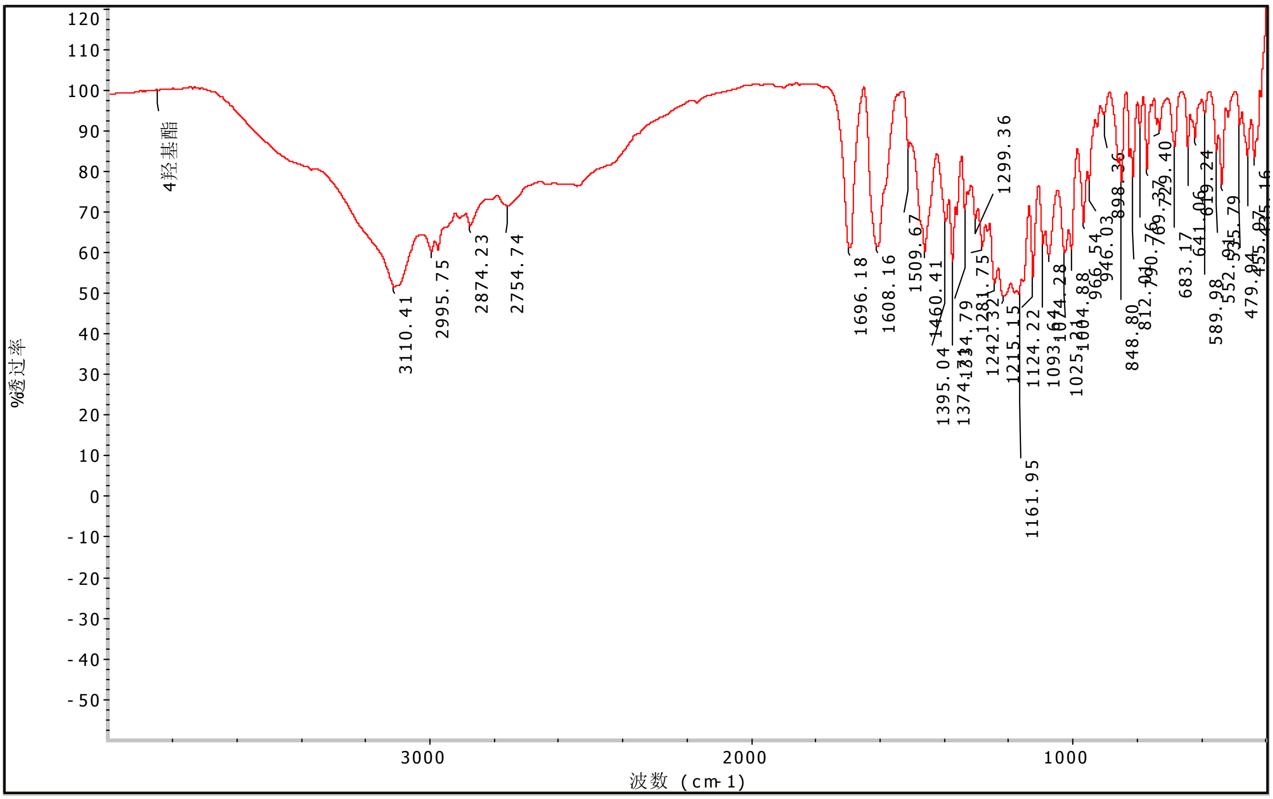

IR spectra of compounds **3k**

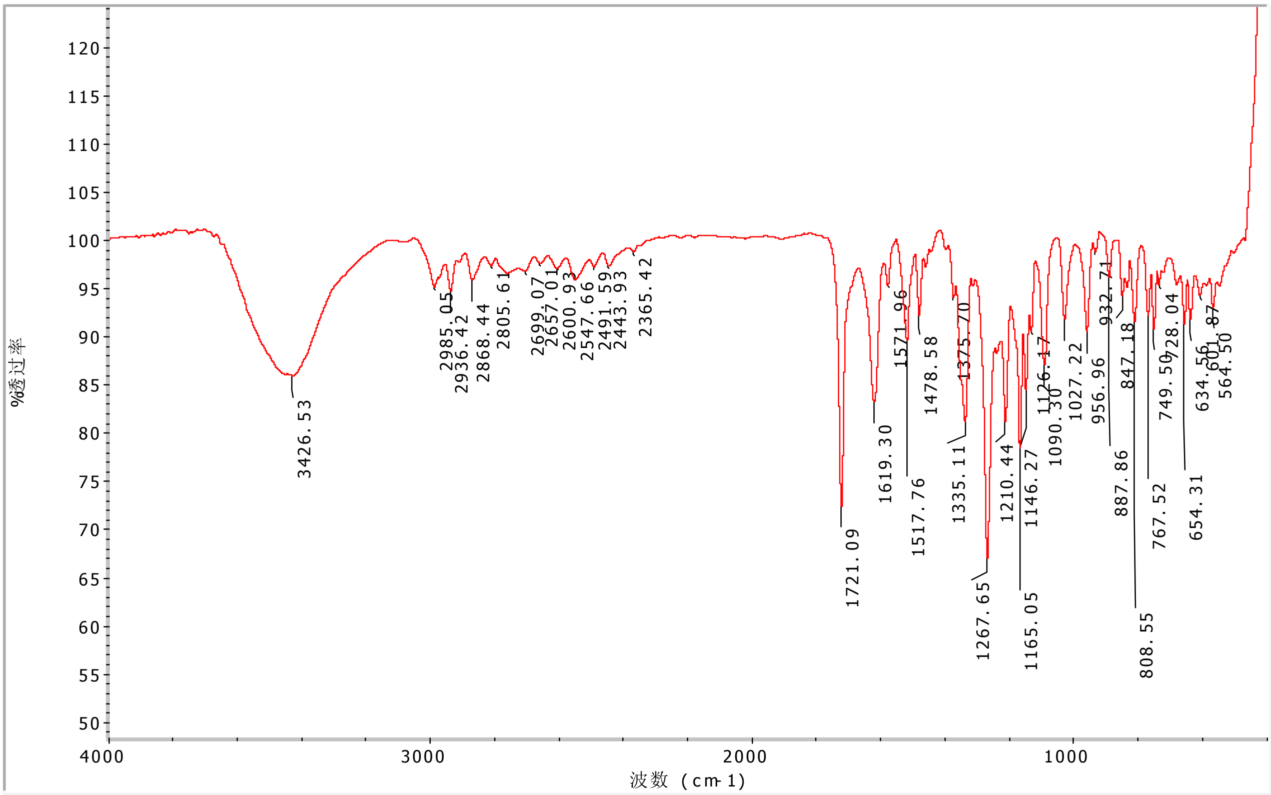

IR spectra of compounds **3l**

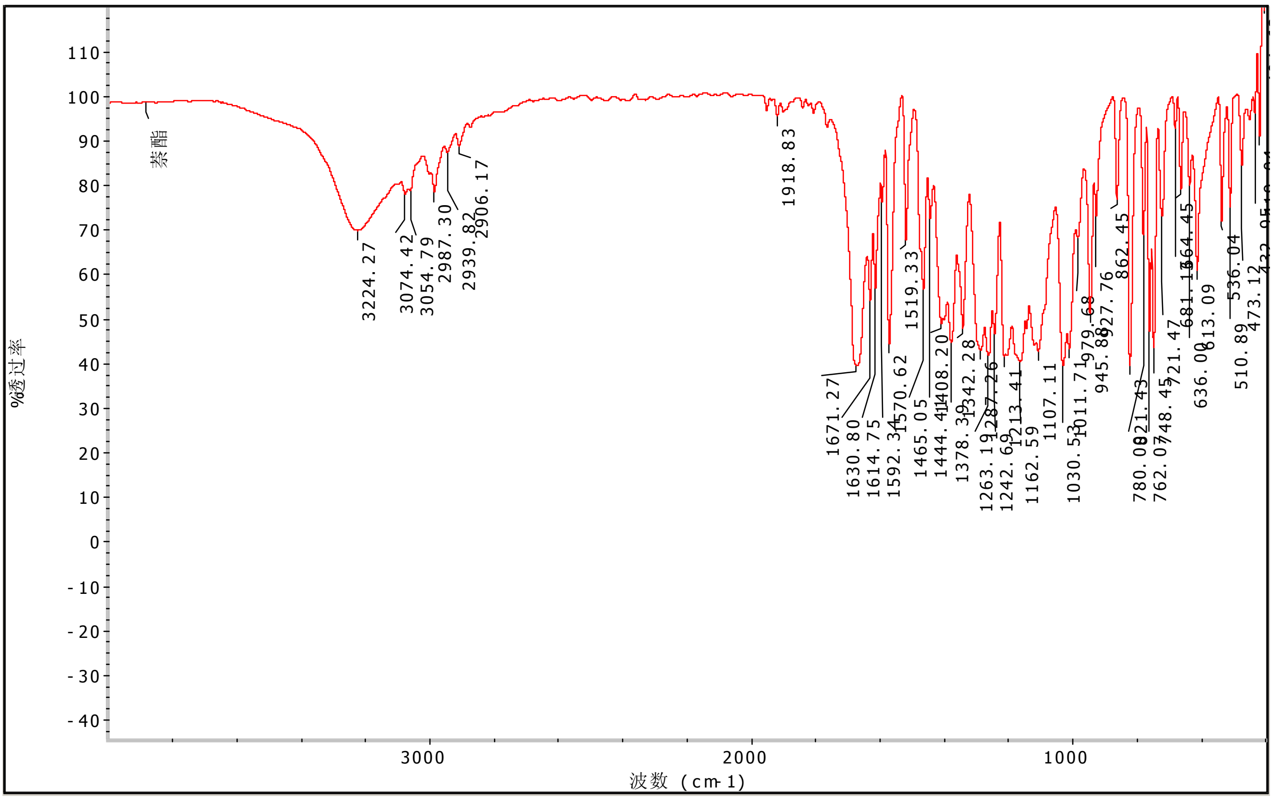

^{1}H-NMR and ^{13}C-NMR of compounds **3a–3l**

^{1}H-NMR and ^{13}C-NMR of compounds **3a**

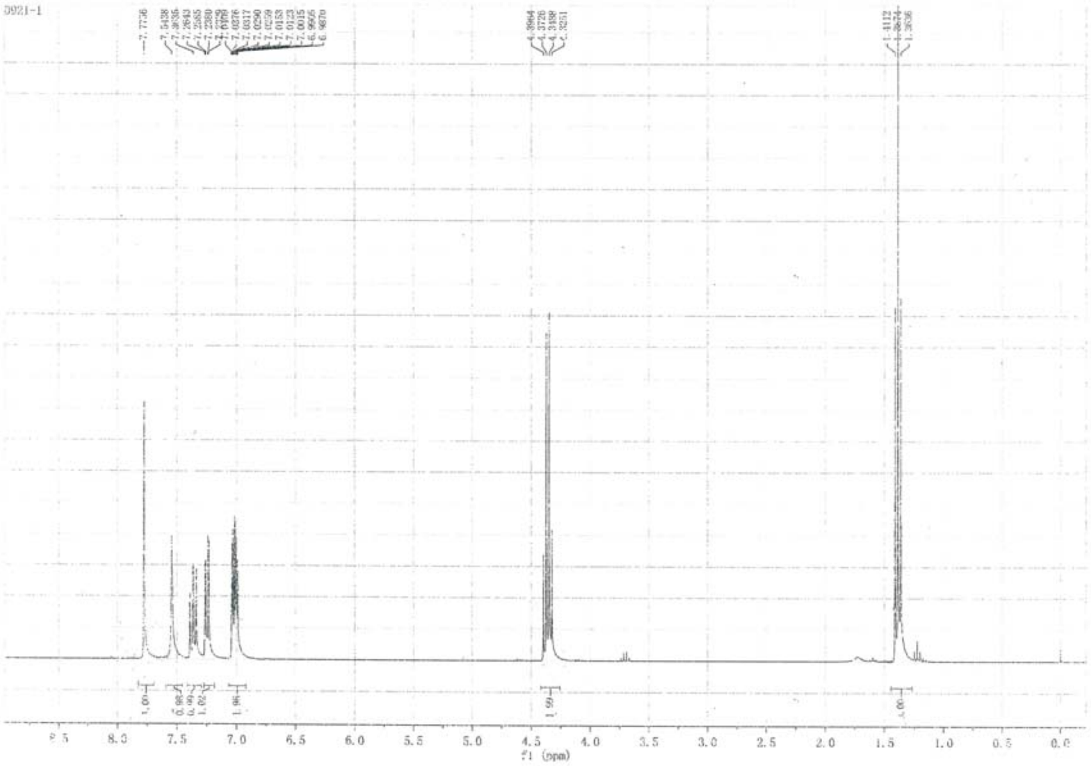

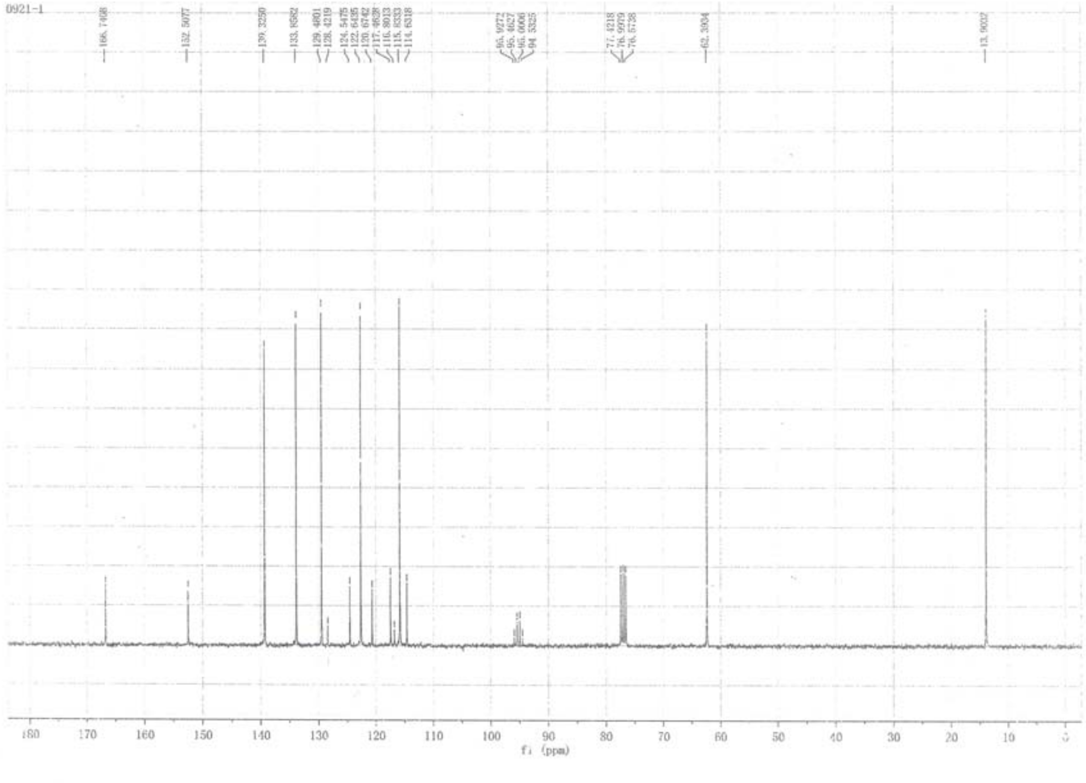

^{1}H-NMR and ^{13}C-NMR of compounds **3b**

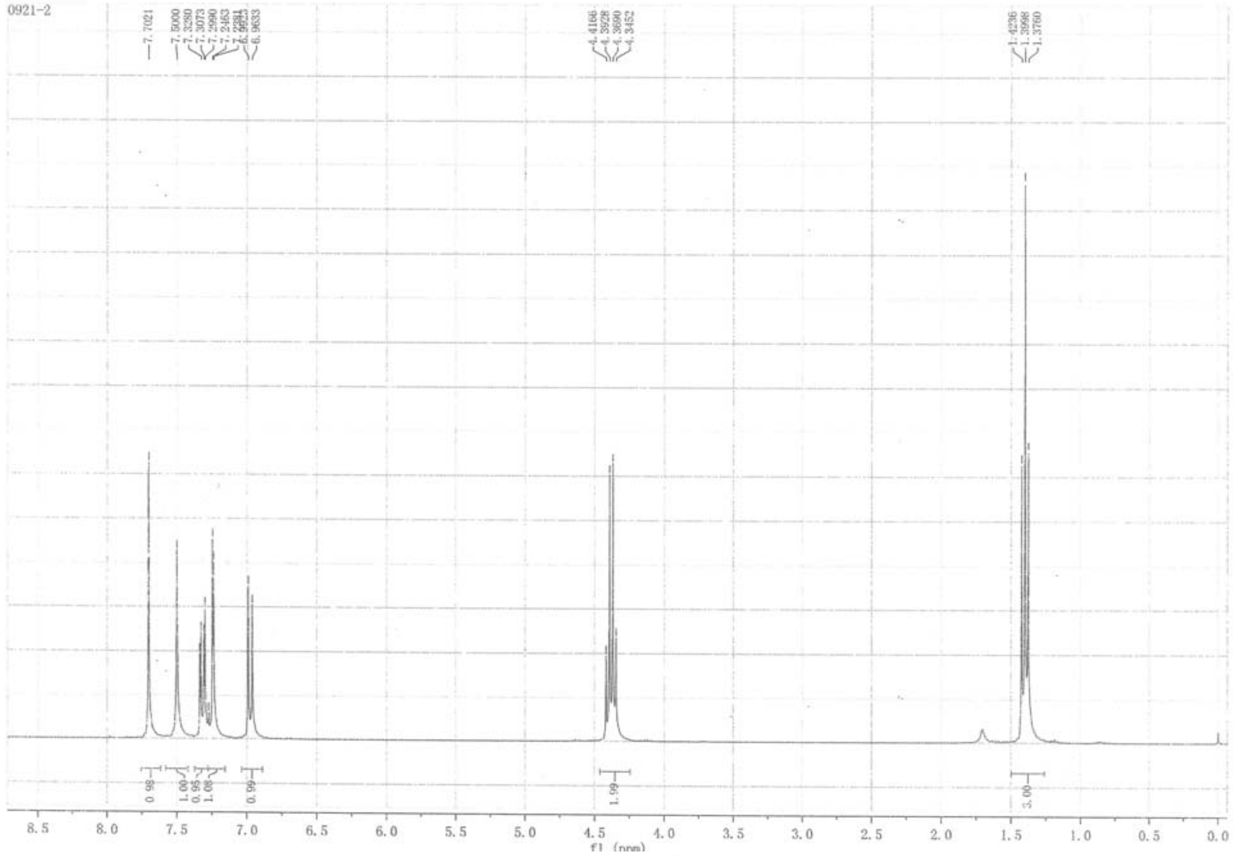

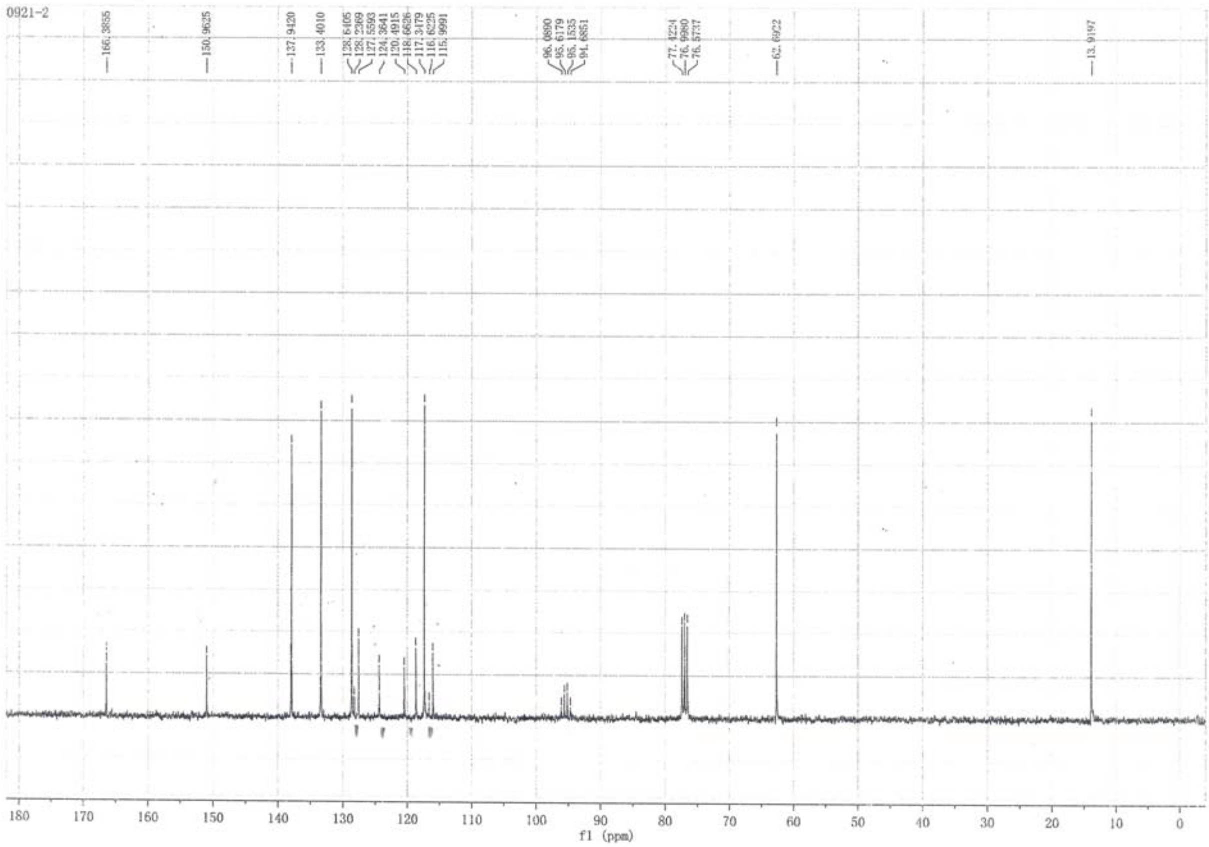

^{1}H-NMR and ^{13}C-NMR of compounds **3c**

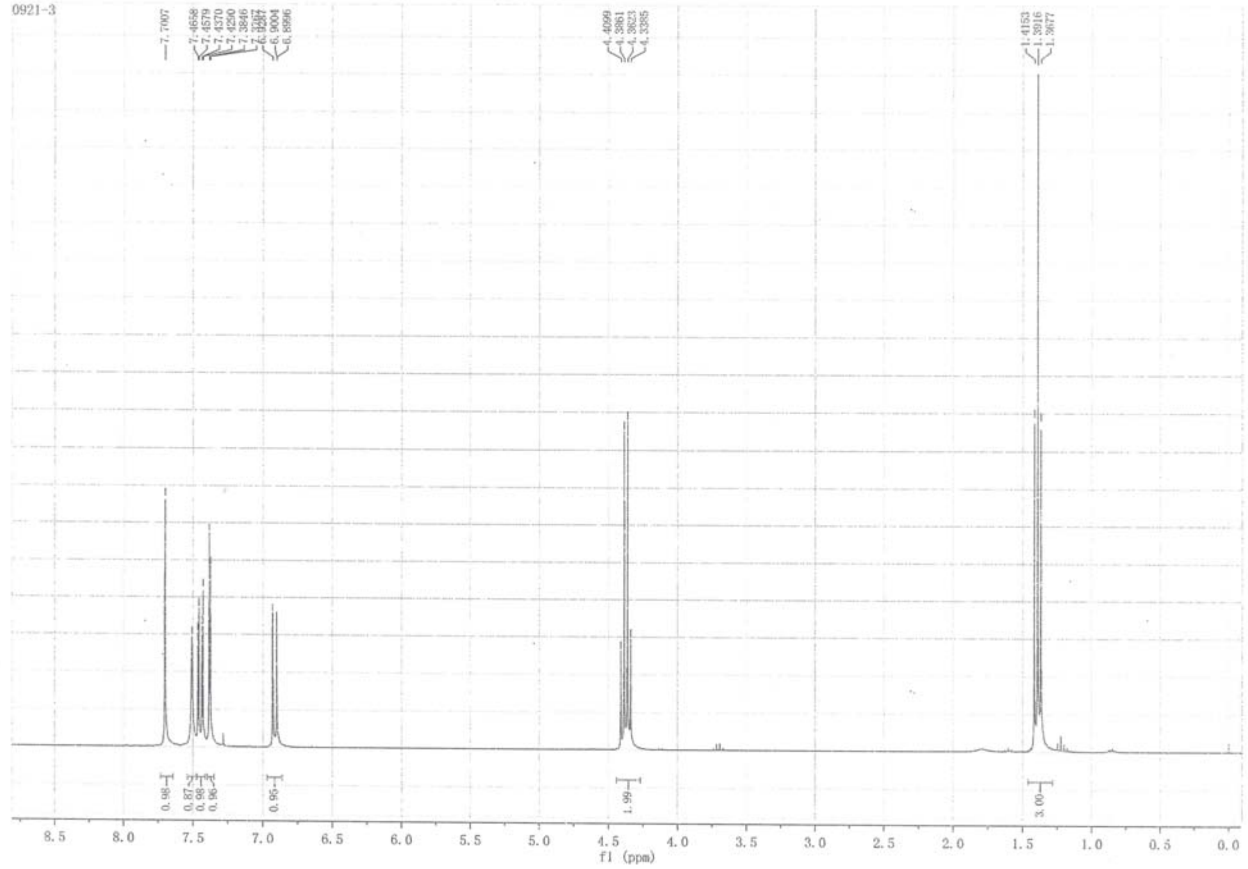

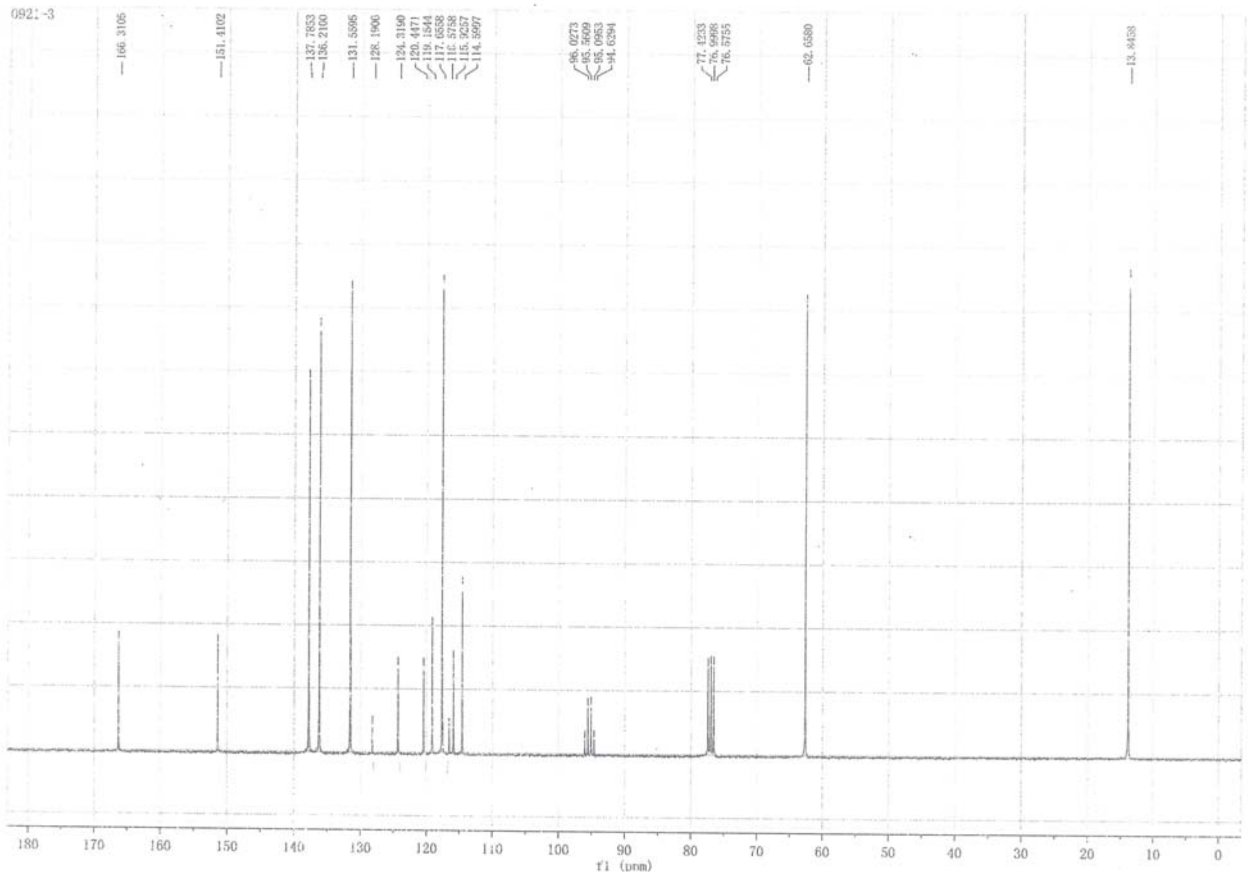

^{1}H-NMR and ^{13}C-NMR of compounds **3d**

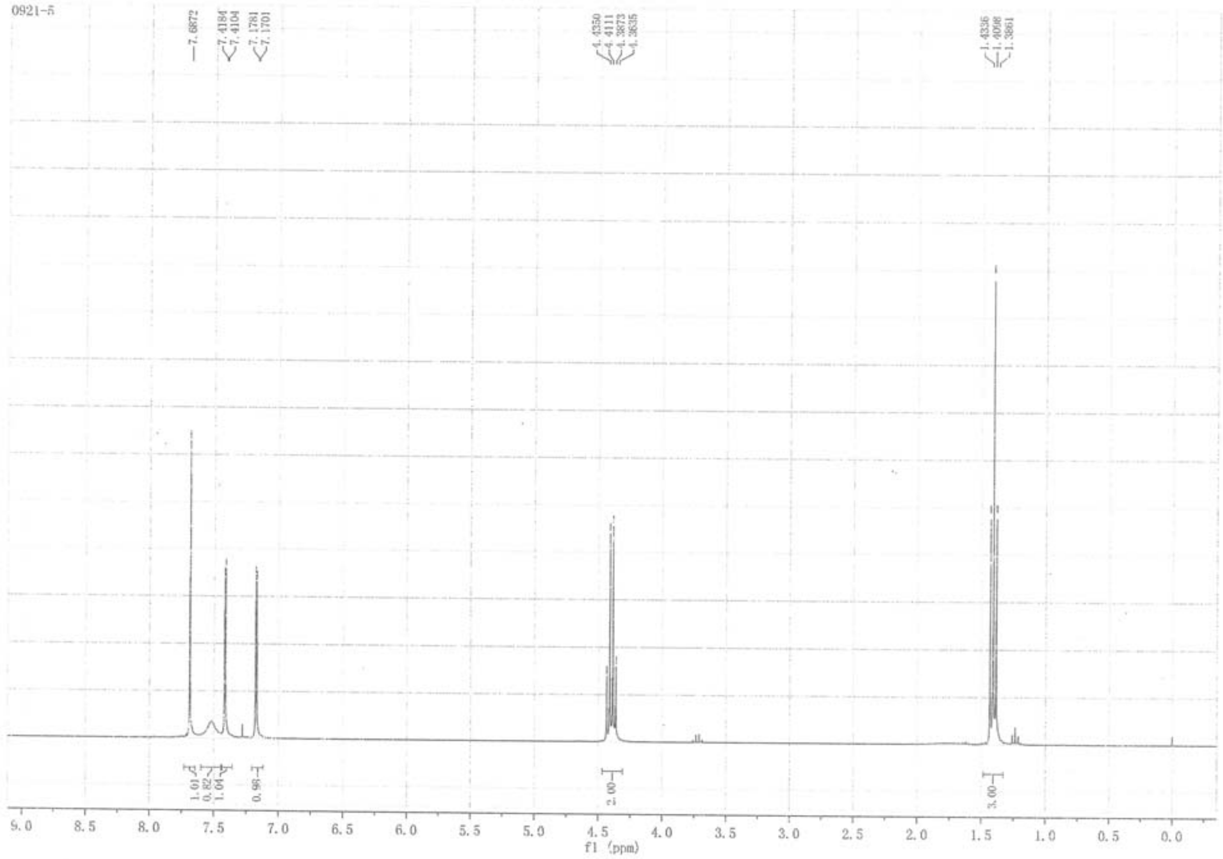

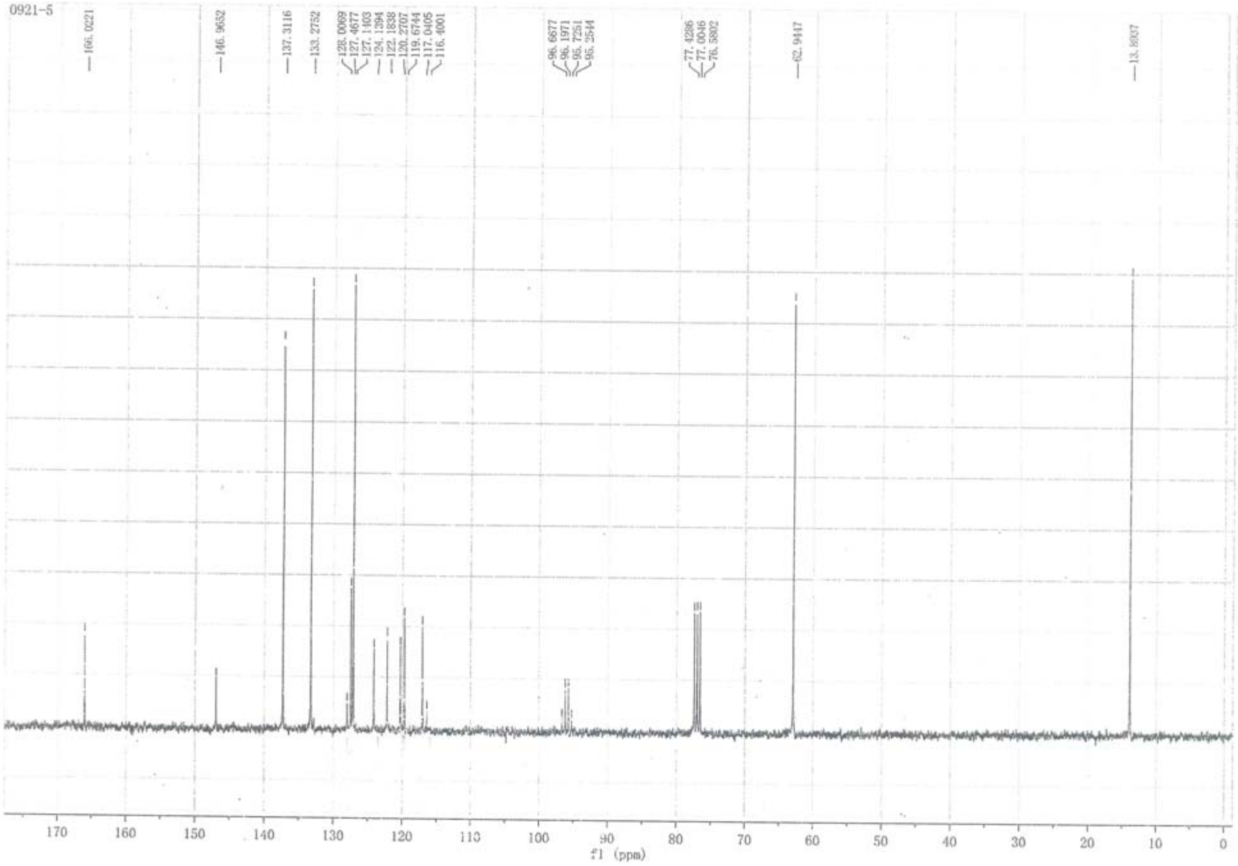

^{1}H-NMR and ^{13}C-NMR of compounds **3e**

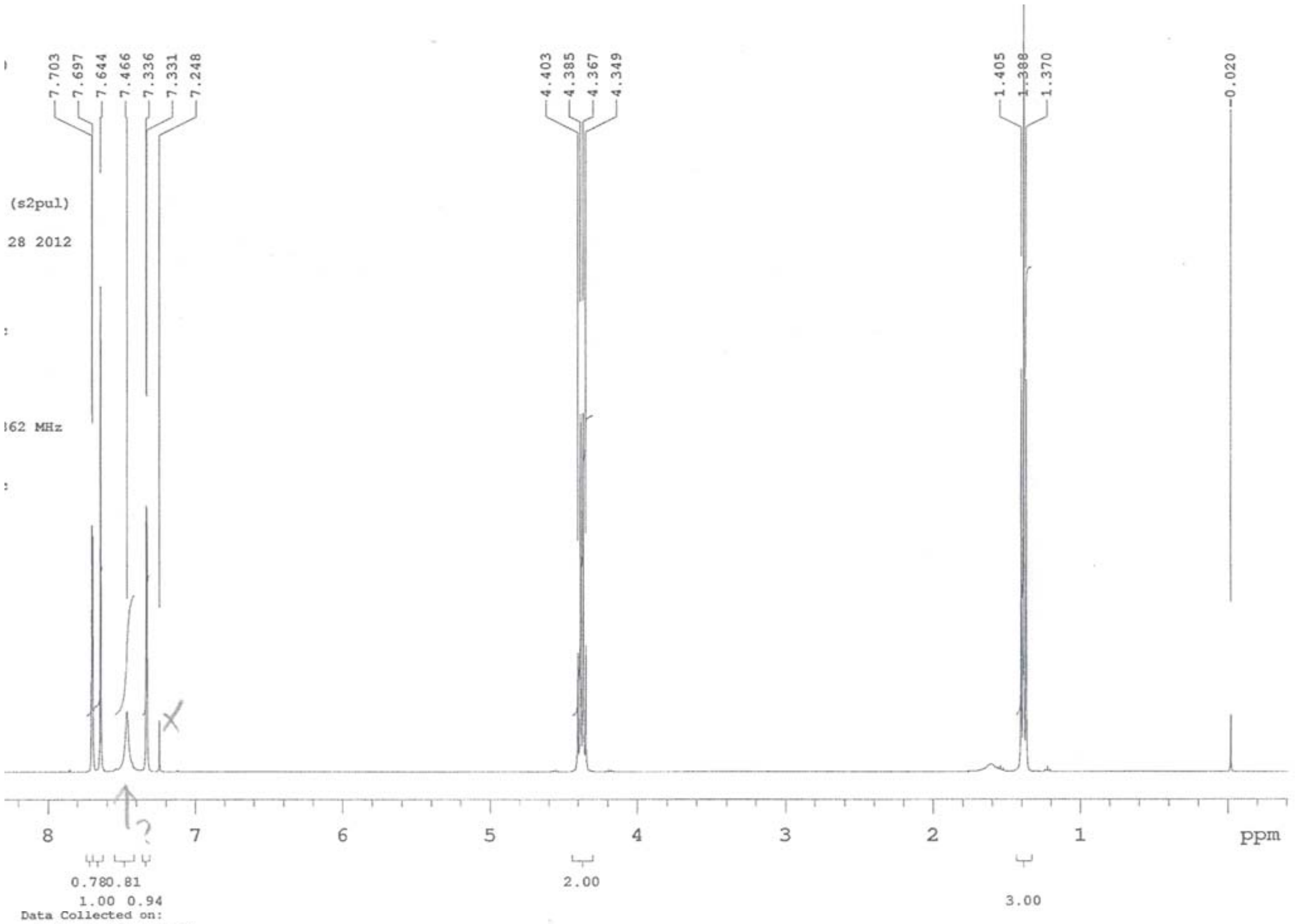

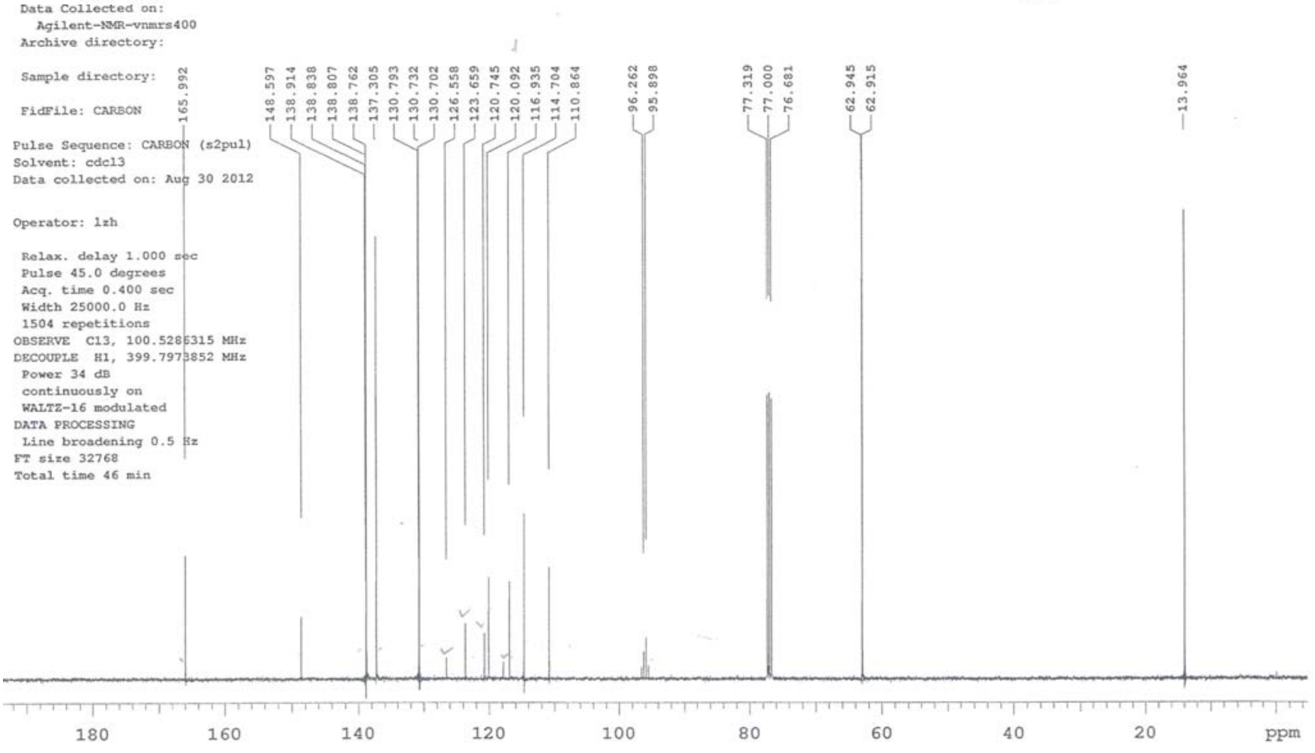

^{1}H-NMR and ^{13}C-NMR of compounds **3f**

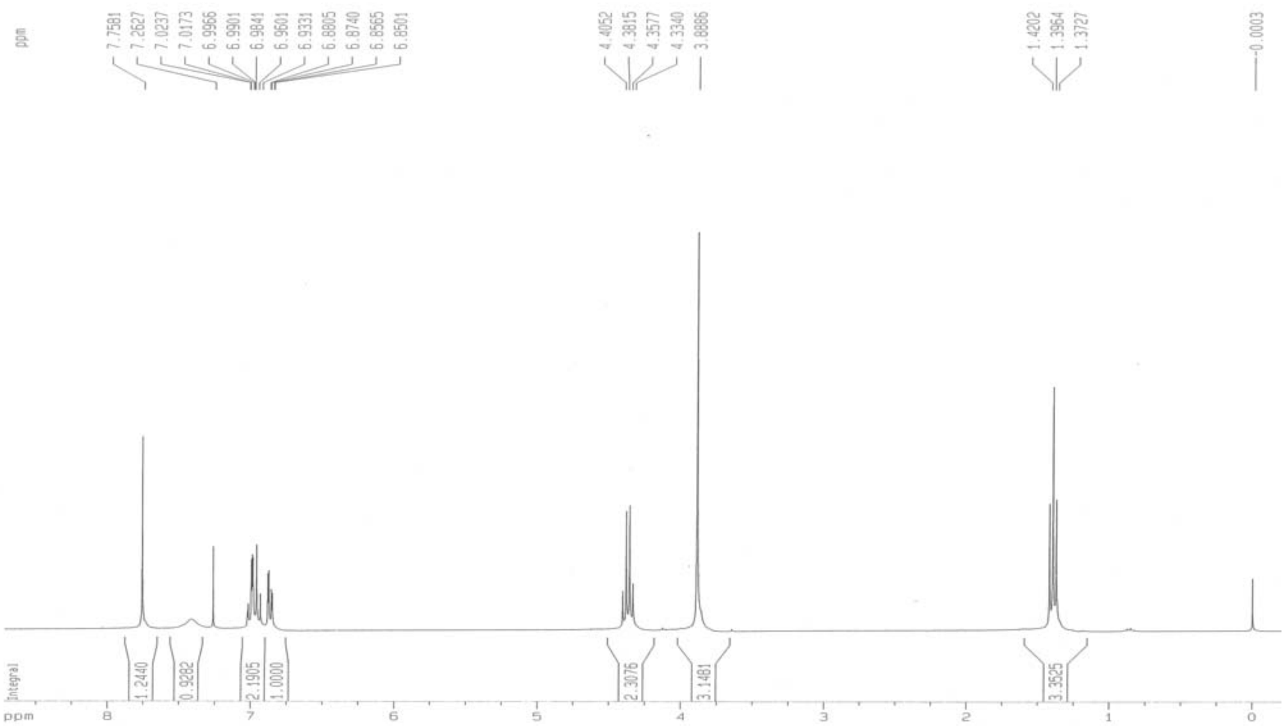

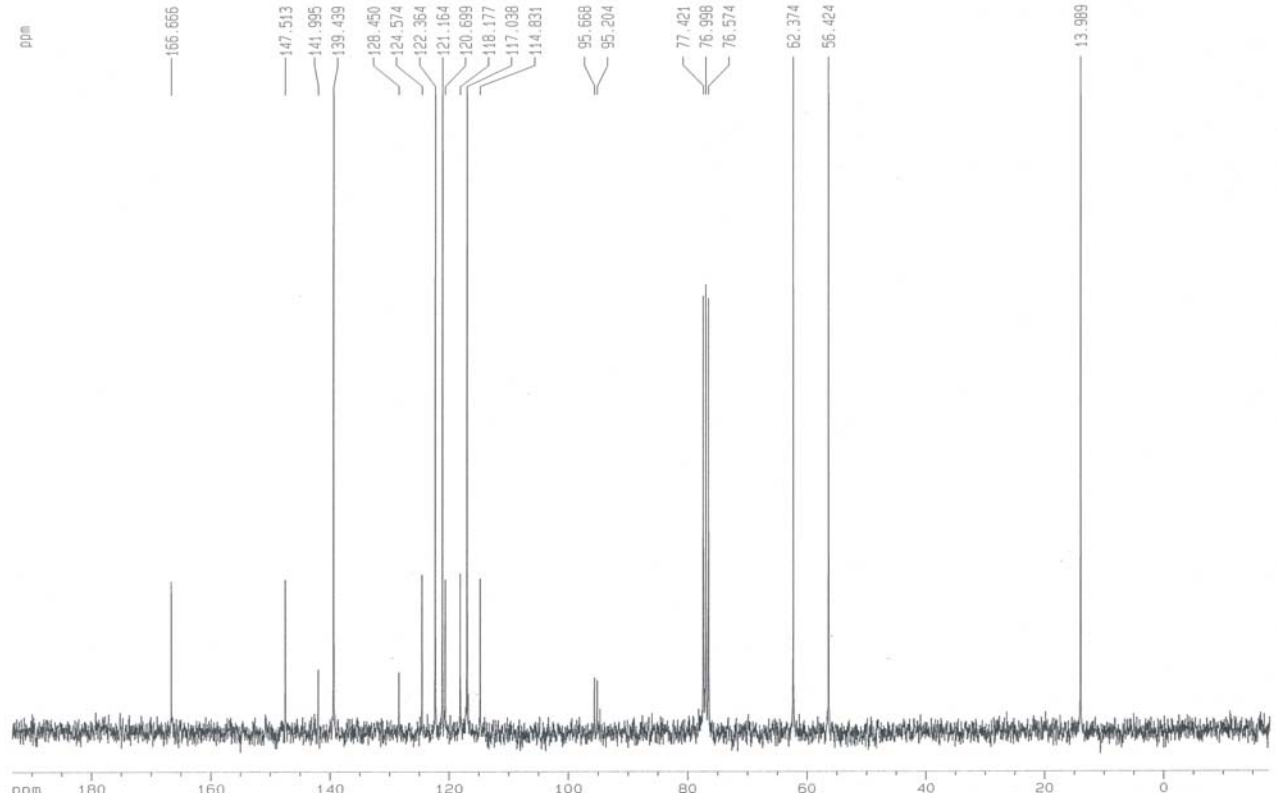

^{1}H-NMR and ^{13}C-NMR of compounds **3g**

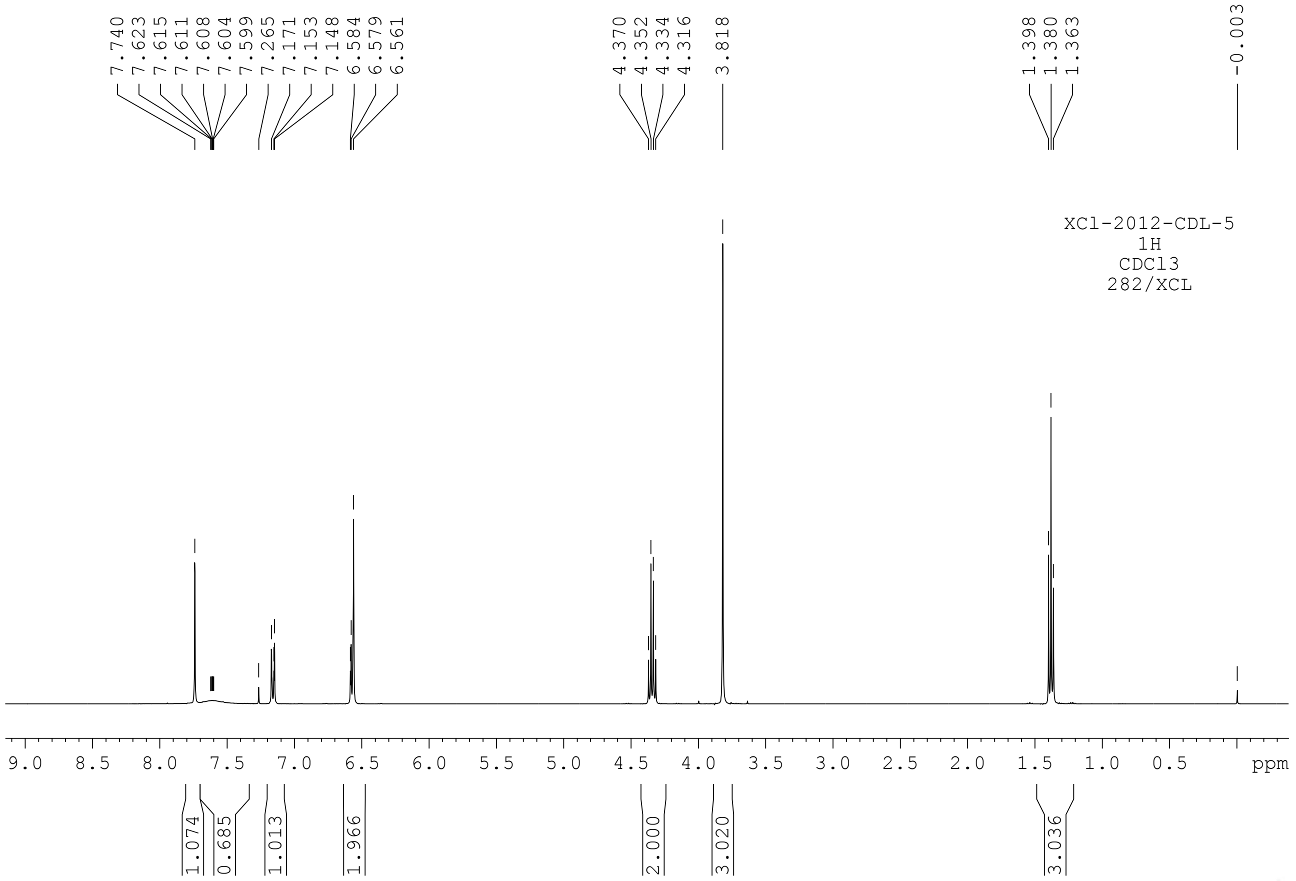

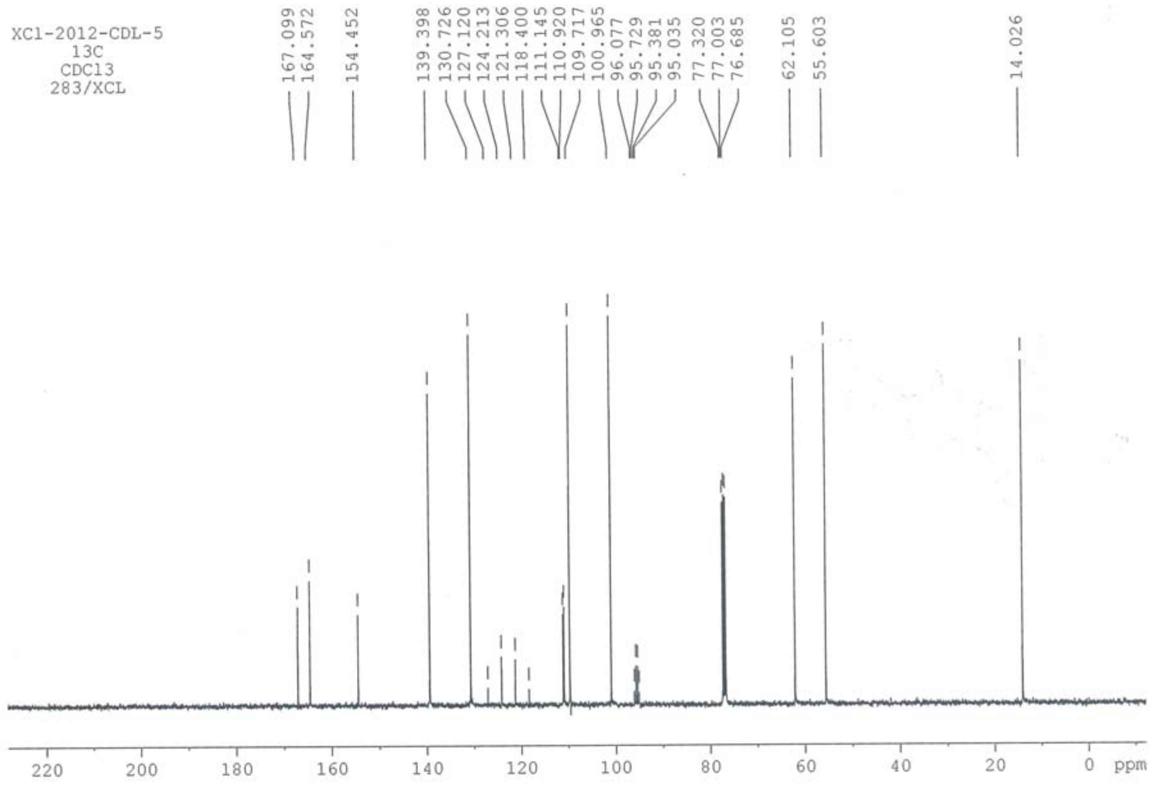

^{1}H-NMR and ^{13}C-NMR of compounds **3h**

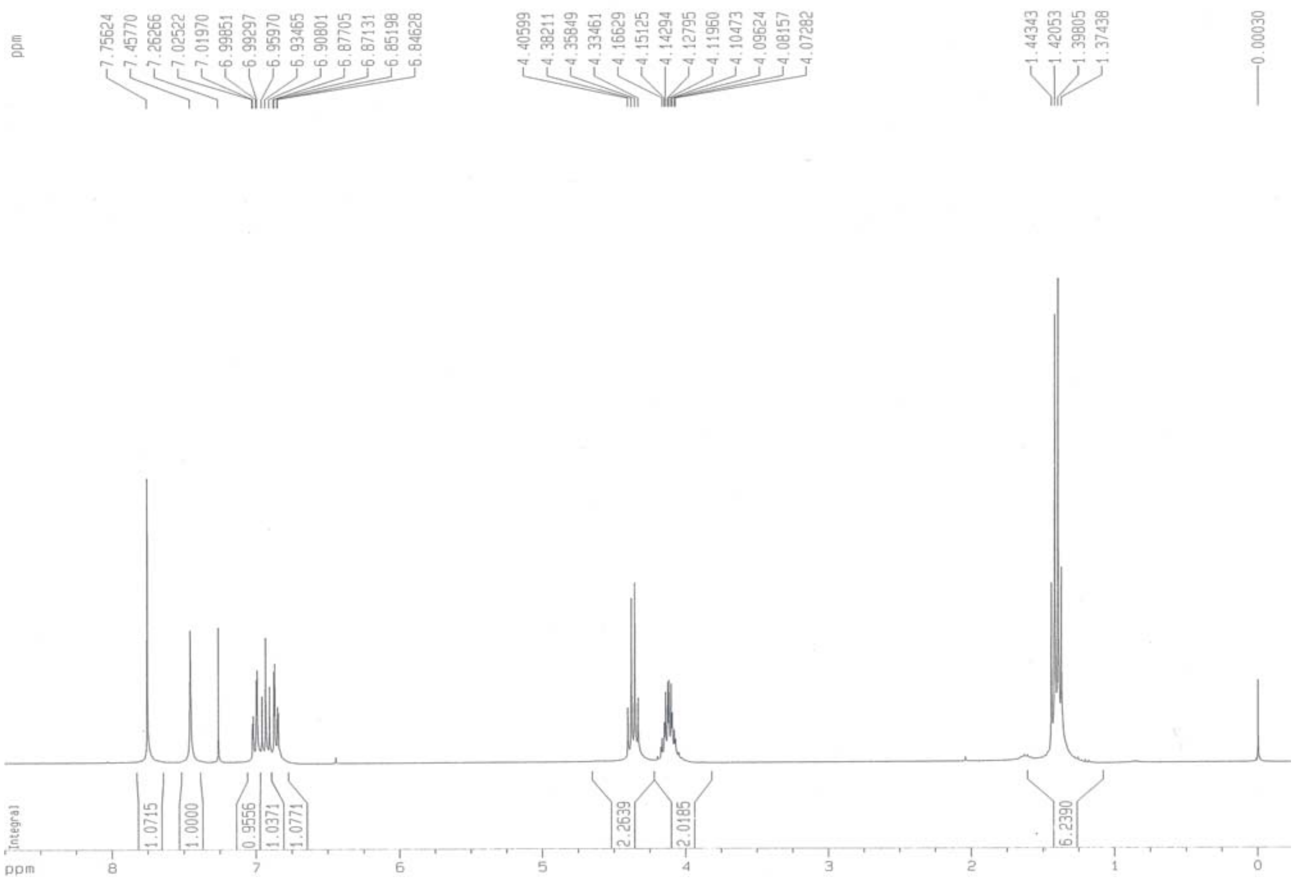

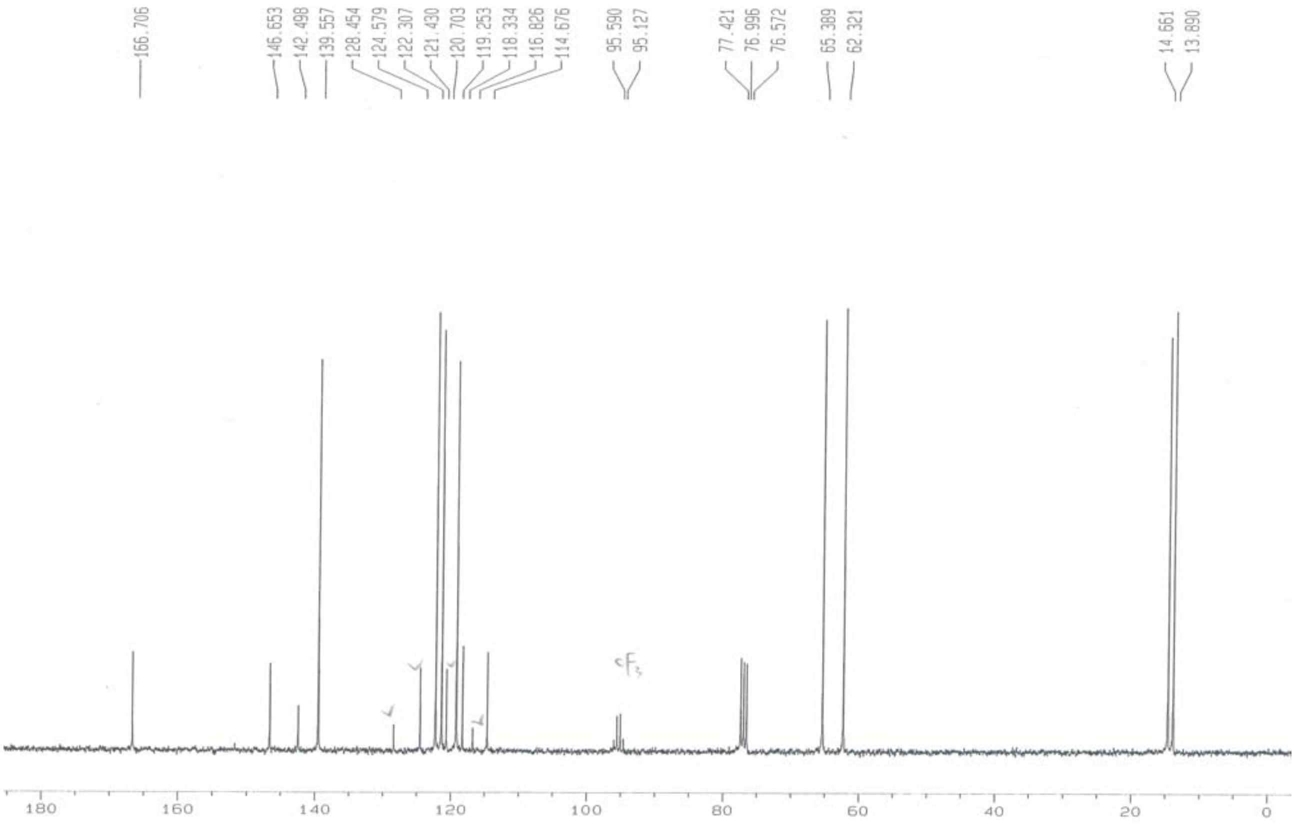

^{1}H-NMR and ^{13}C-NMR of compounds **3i**

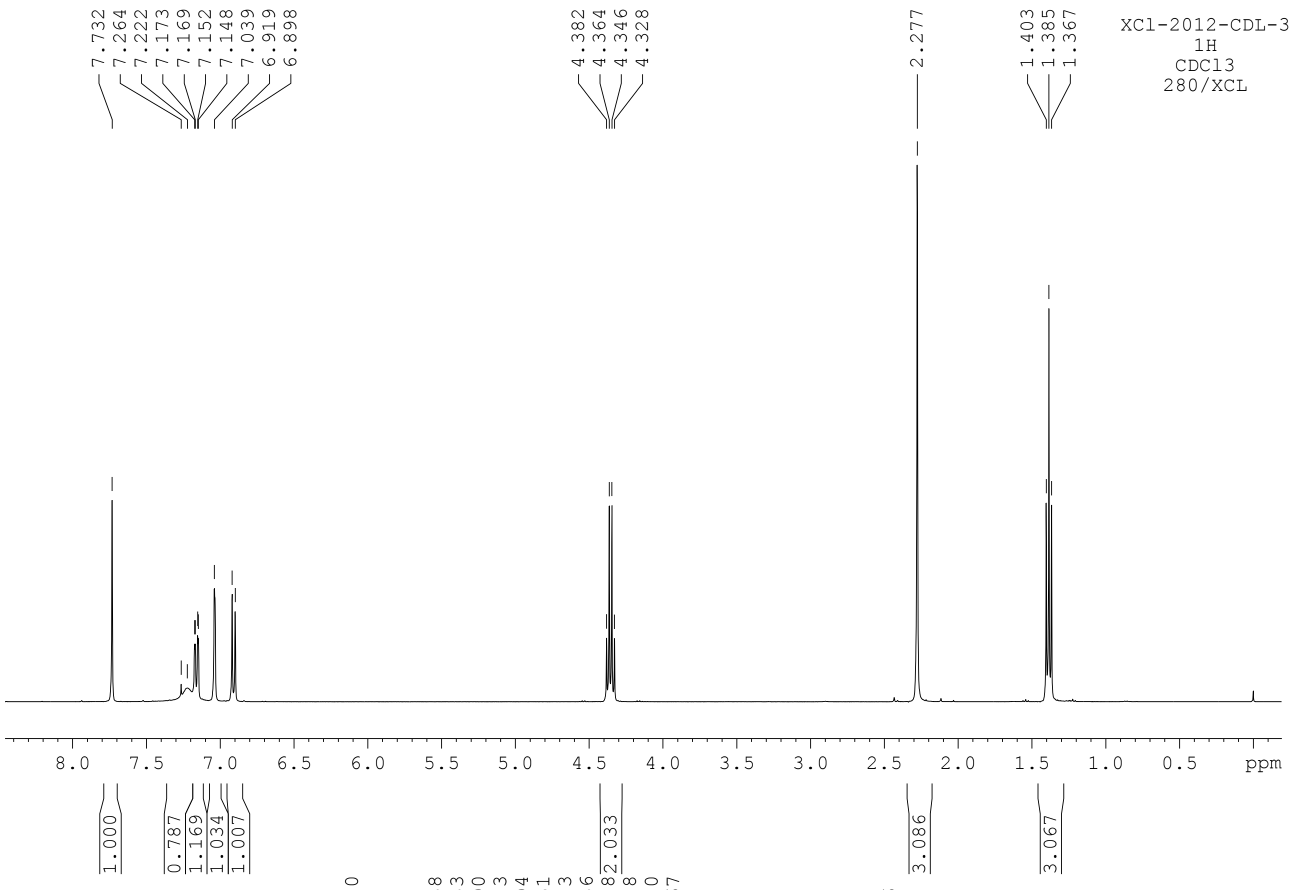

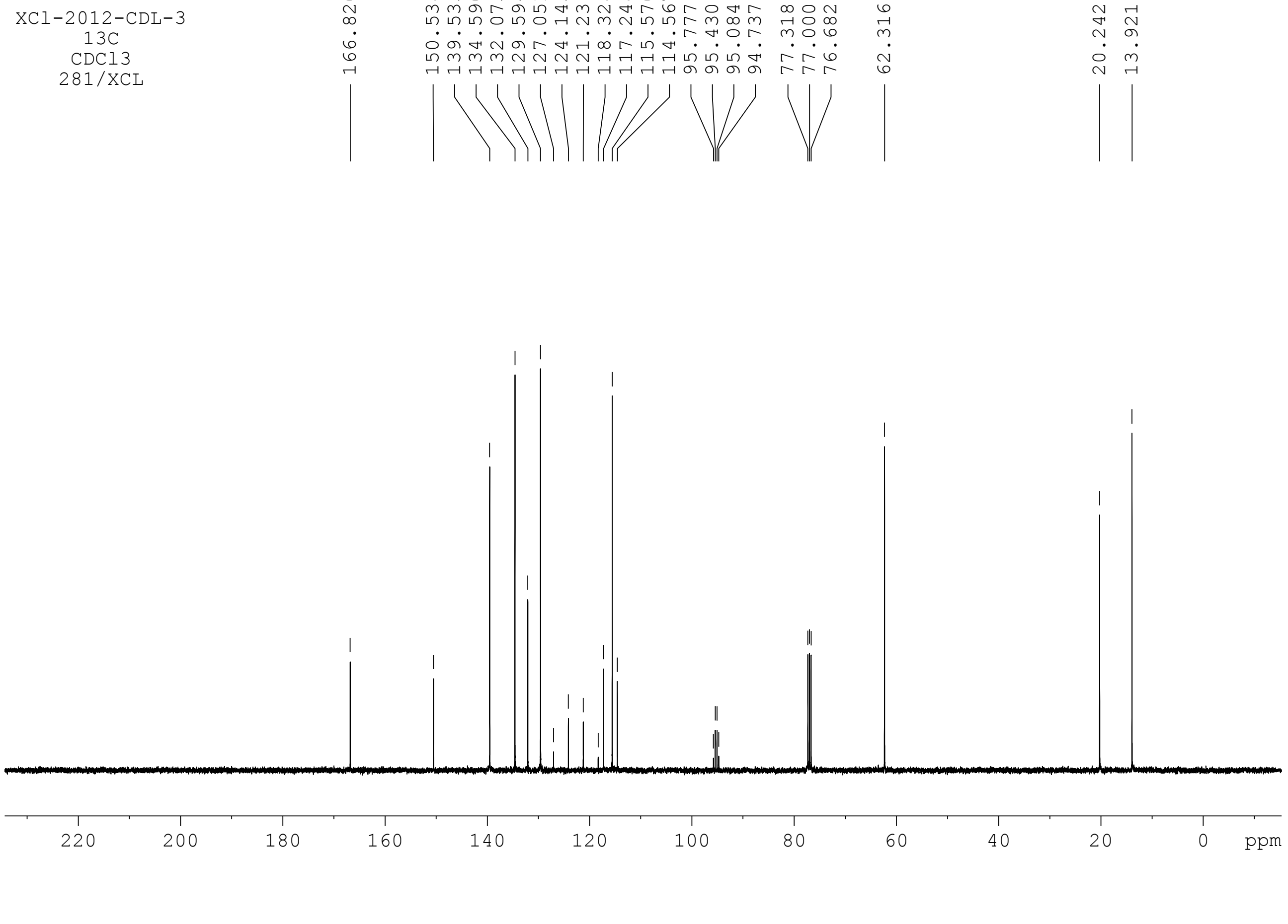

^{1}H-NMR and ^{13}C-NMR of compounds **3j**

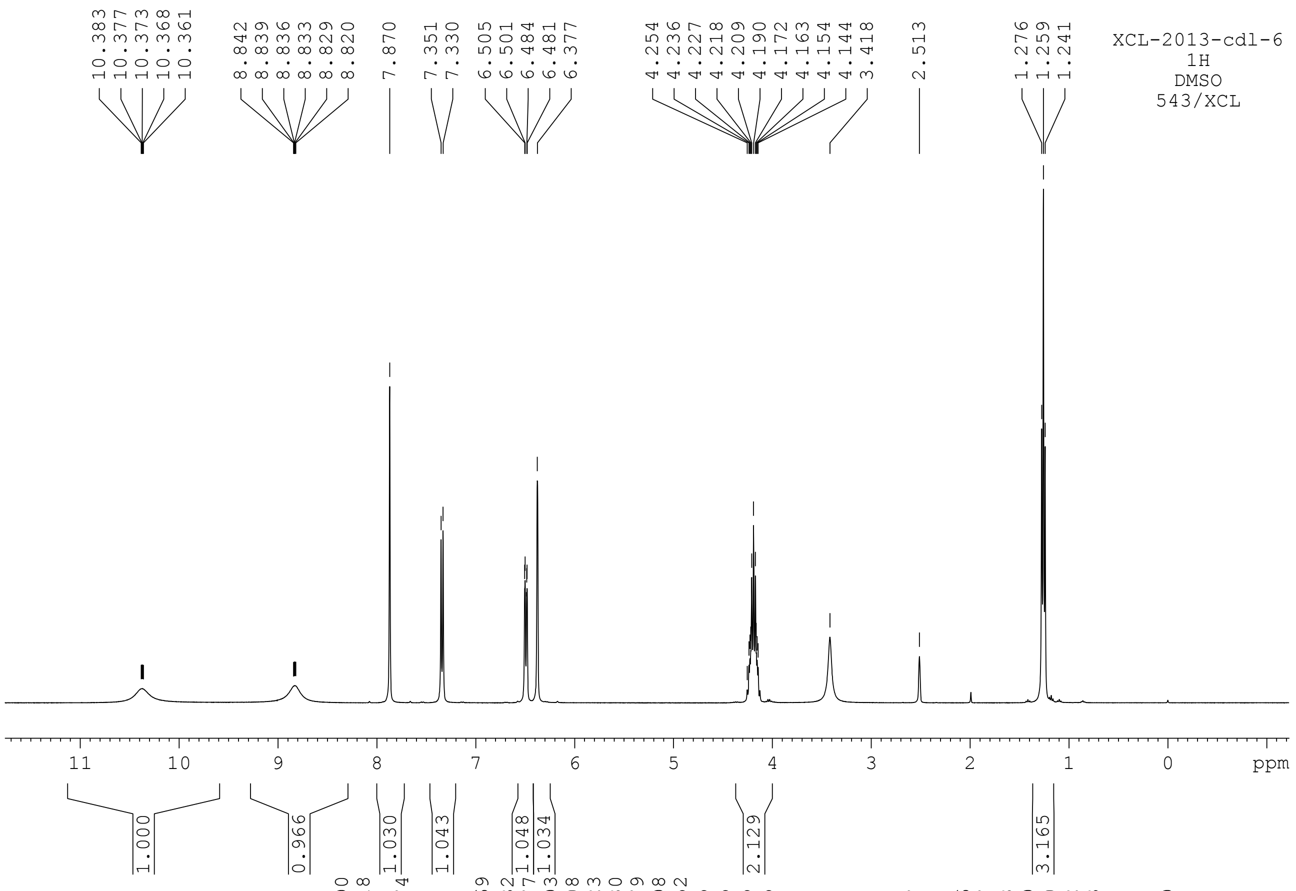

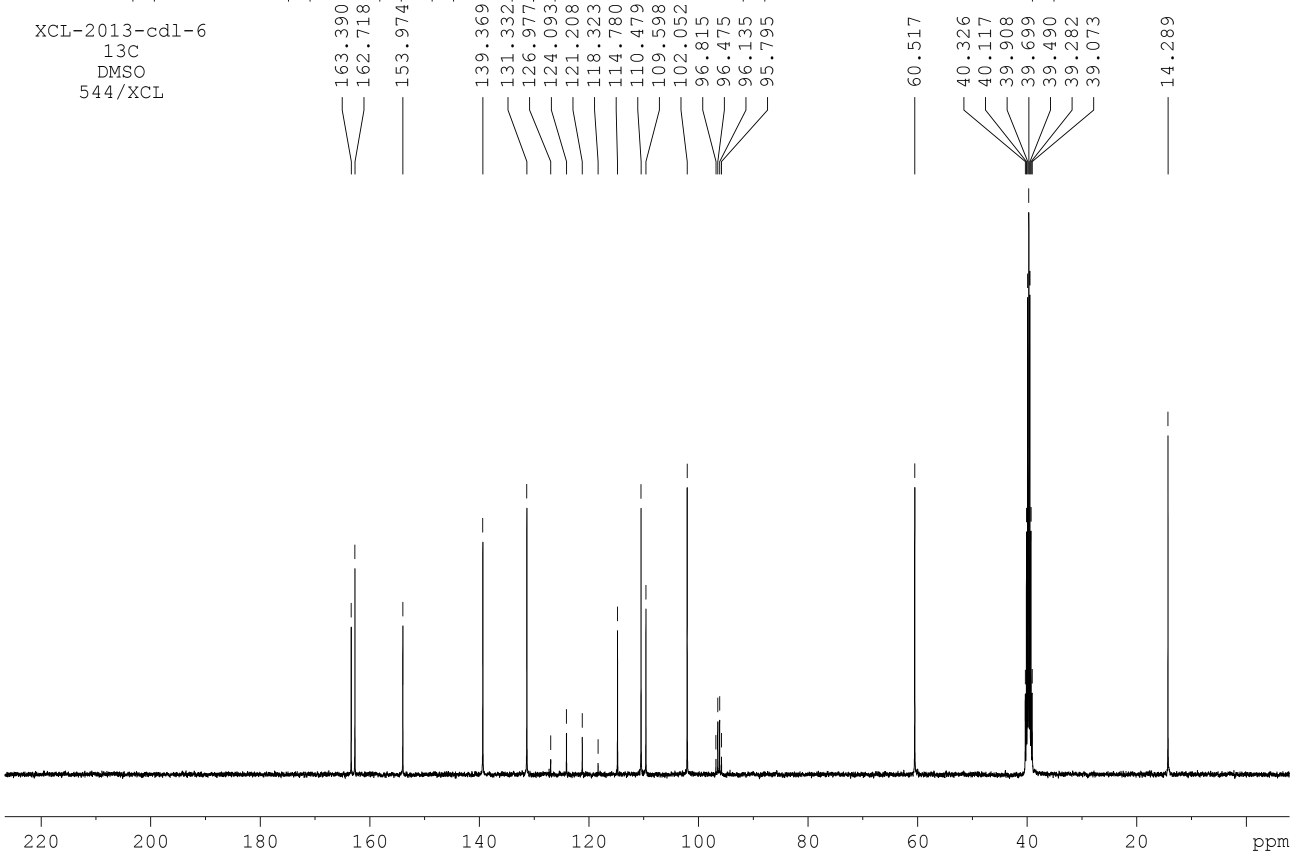

^{1}H-NMR and ^{13}C-NMR of compounds **3k**

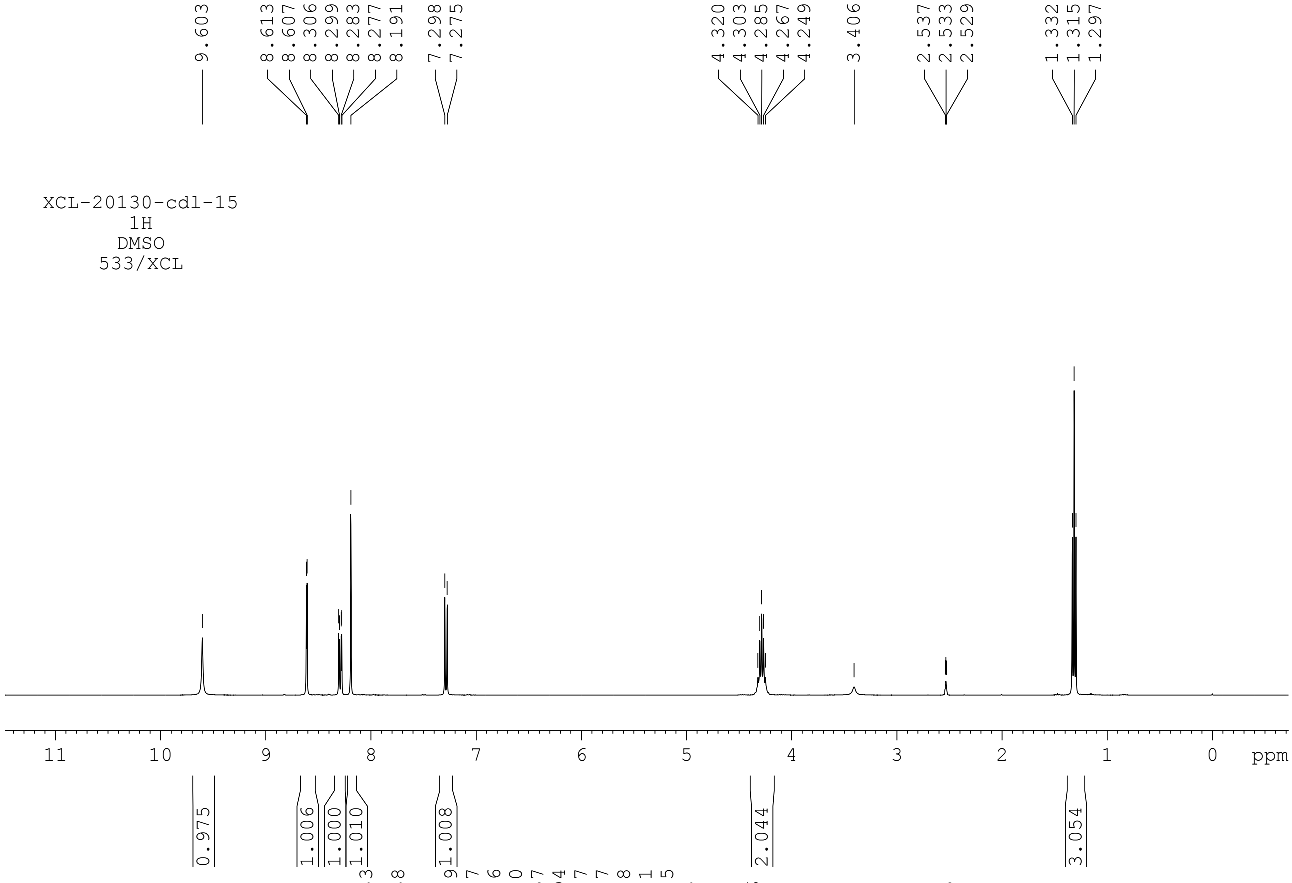

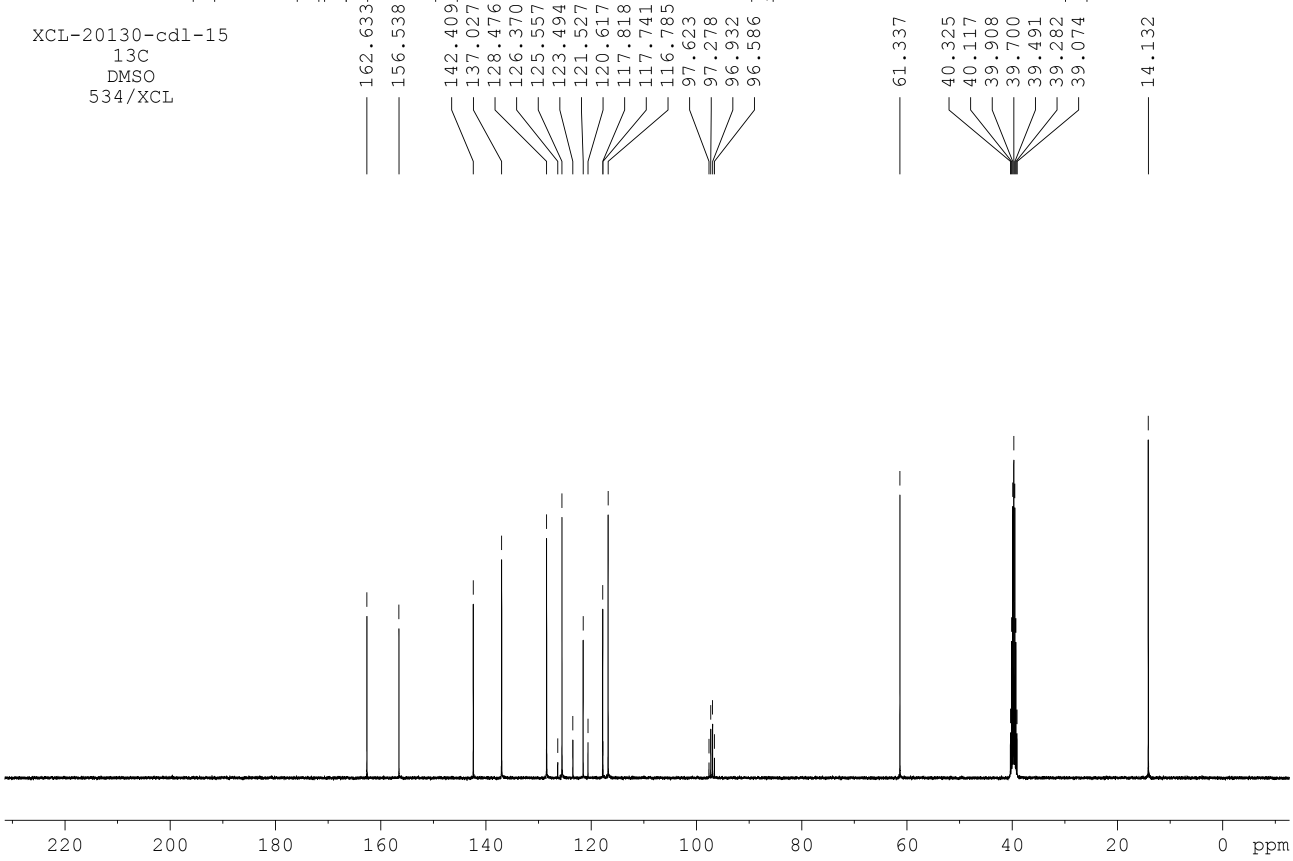

^{1}H-NMR and ^{13}C-NMR of compounds **3l**

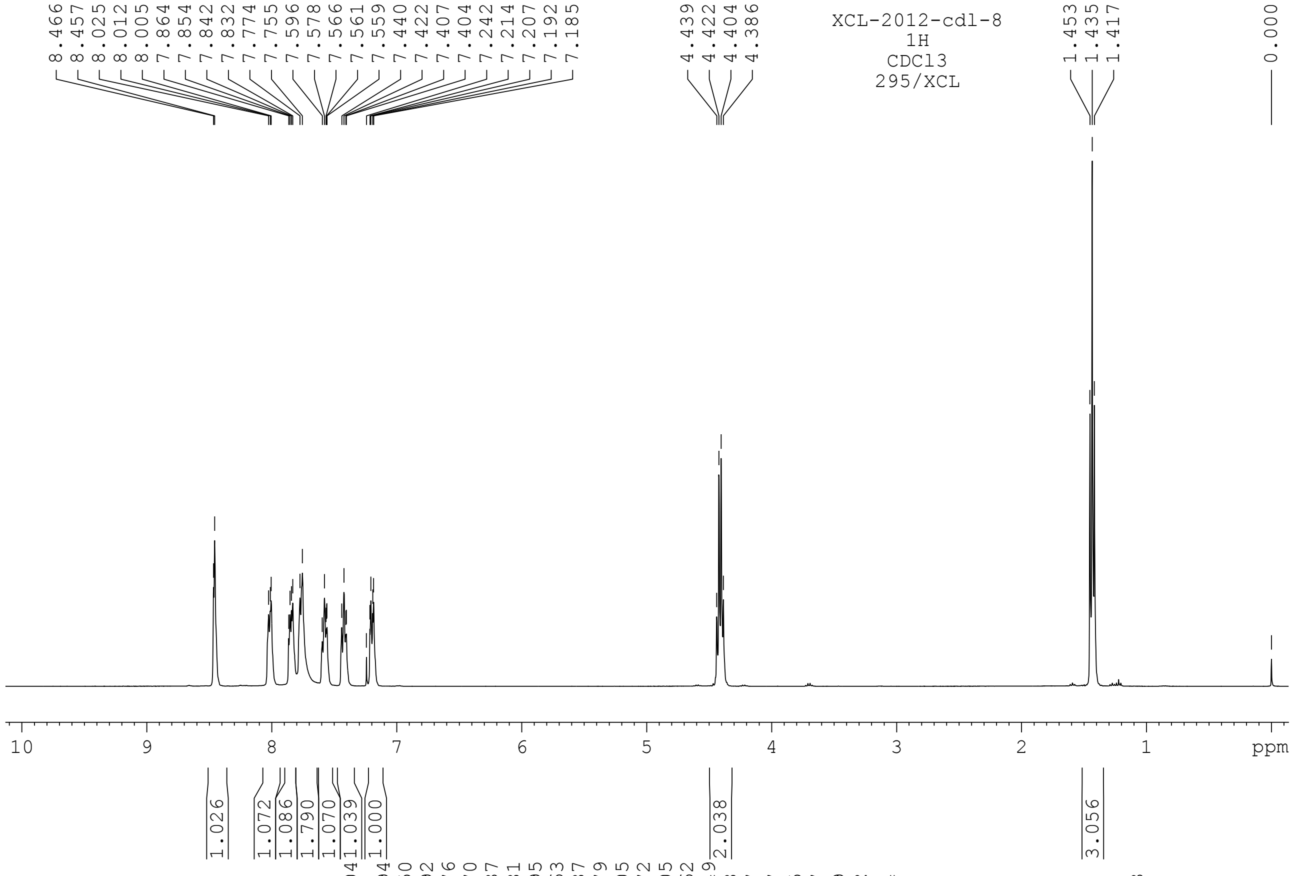

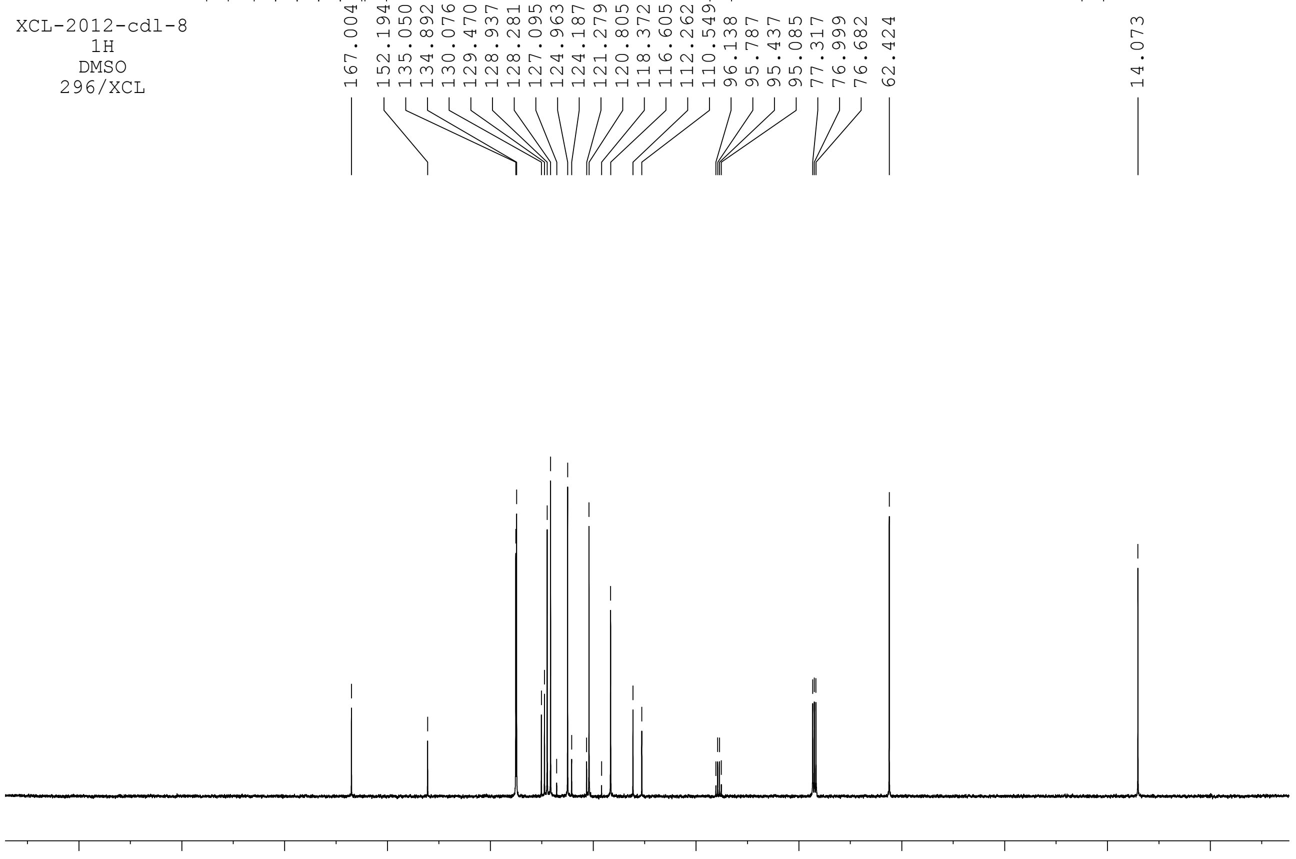

^{19}F-NMR of compounds **3d**

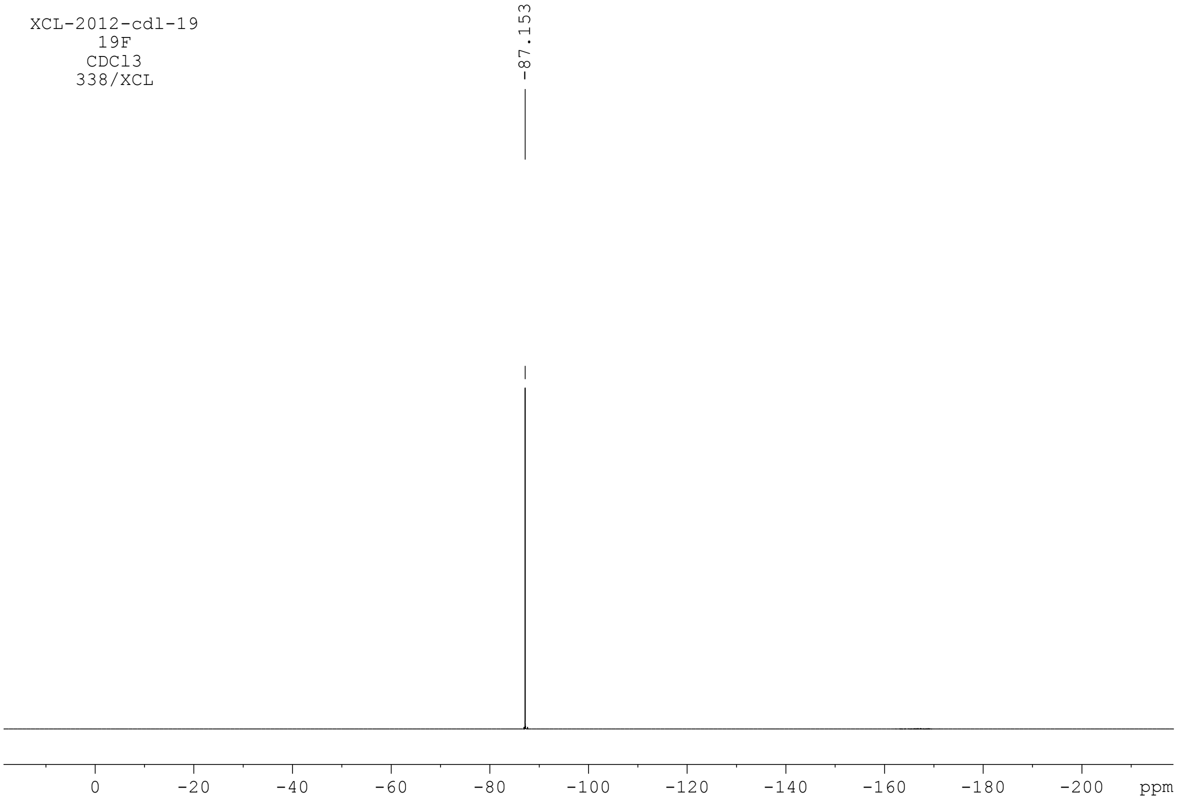

HRMS of compounds **3a-3l**

HRMS of compounds **3a**

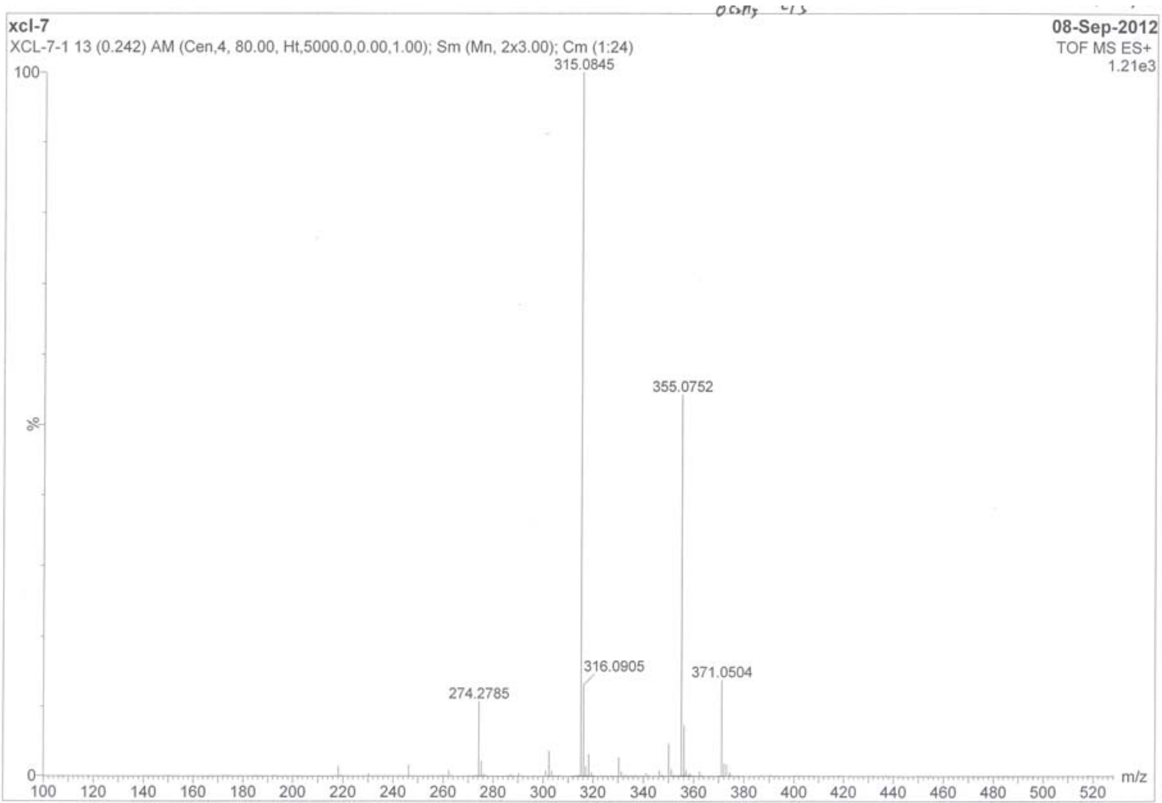

HRMS of compounds **3b**

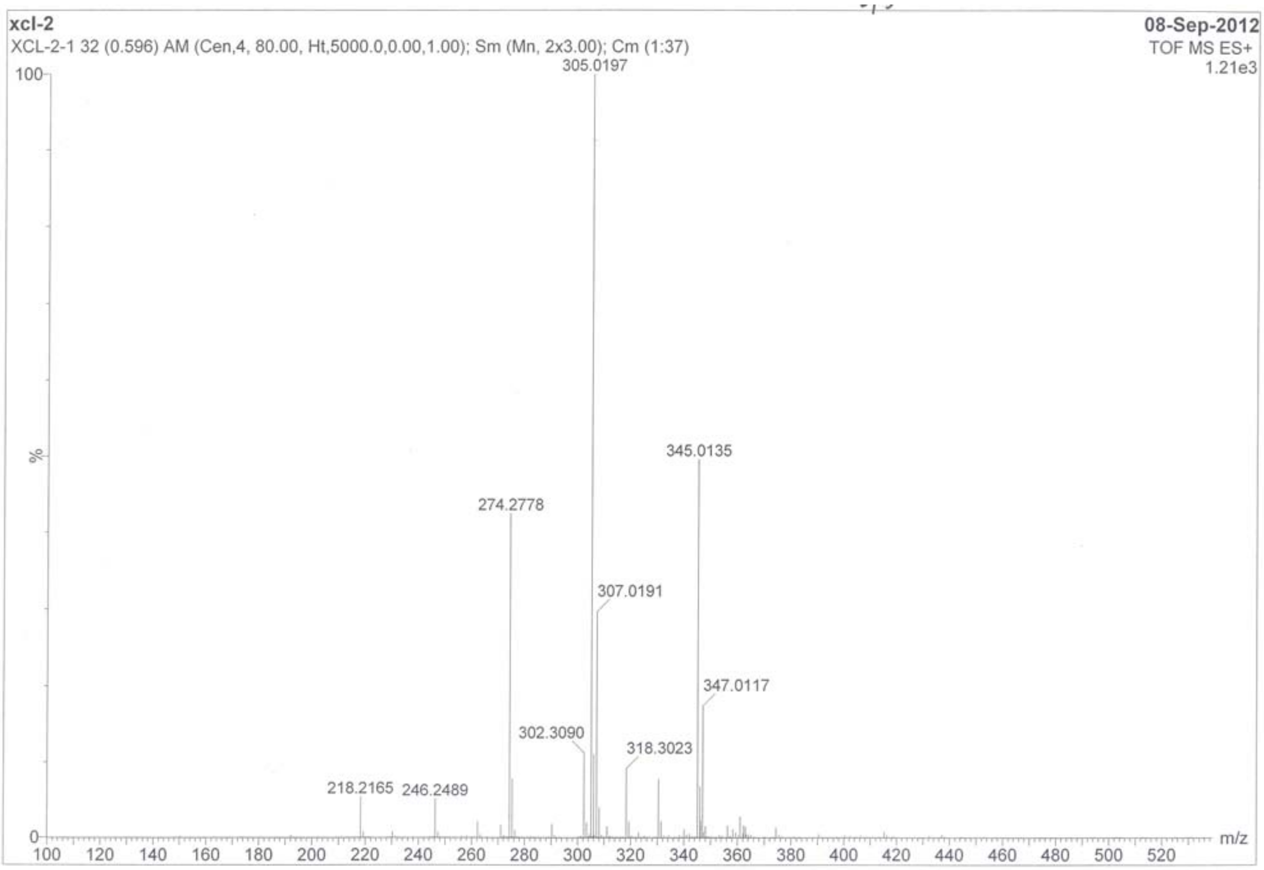

HRMS of compounds **3c**

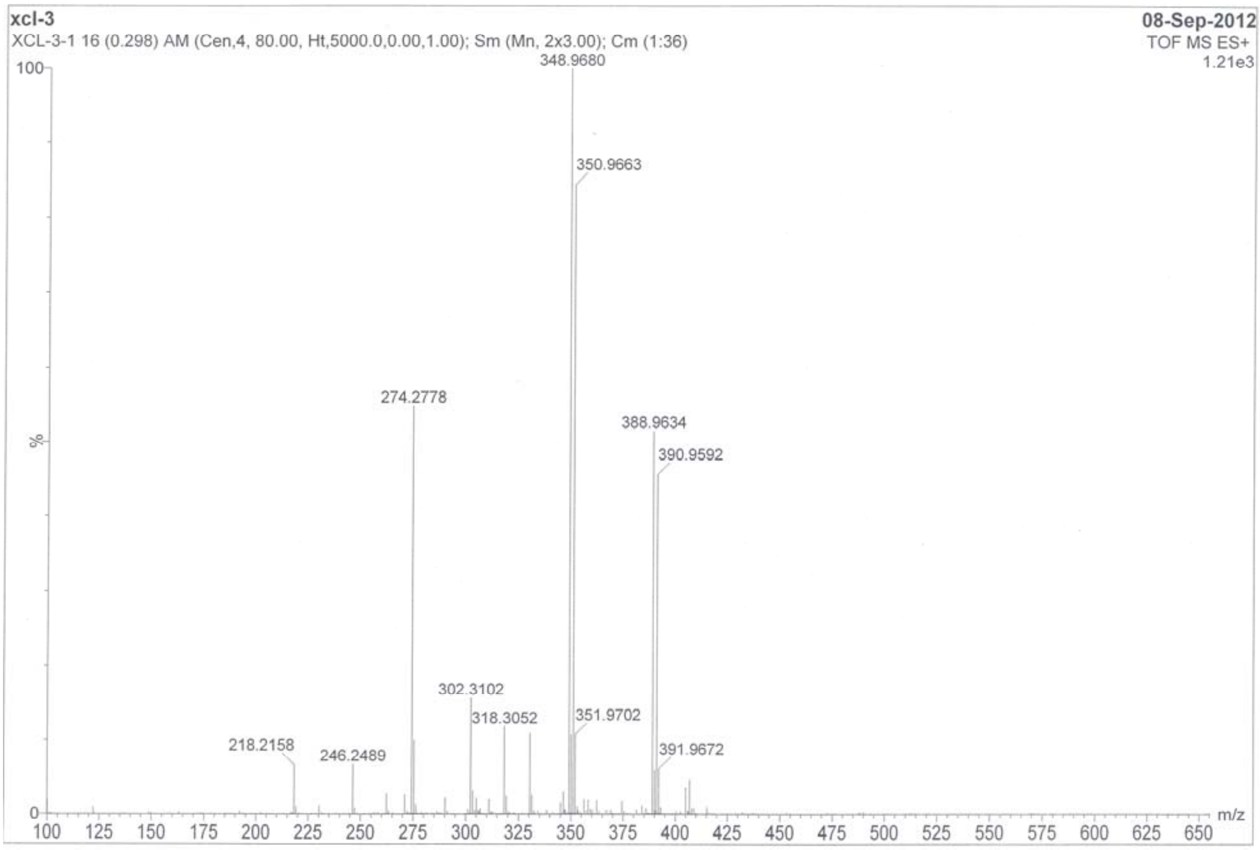

HRMS of compounds **3d**

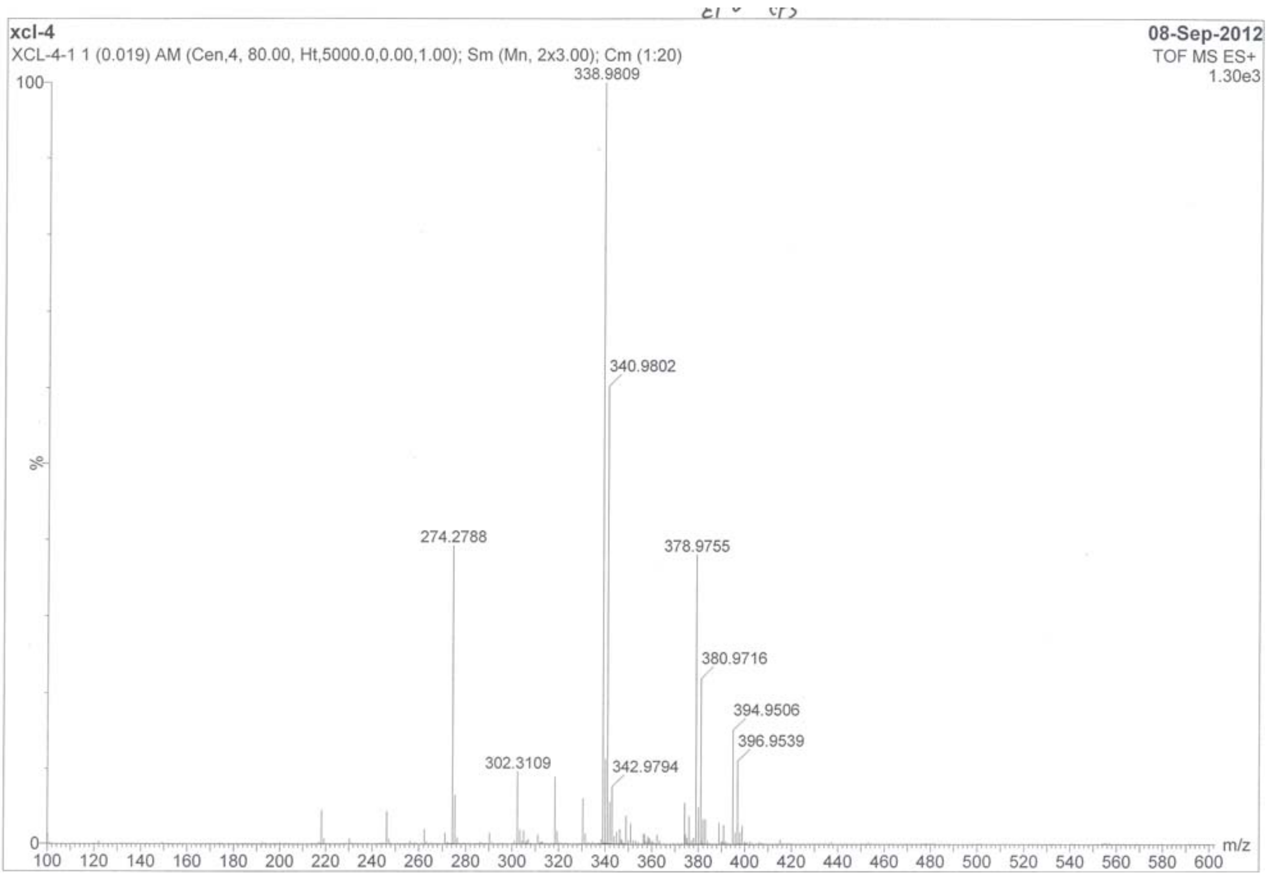

HRMS of compounds **3e**

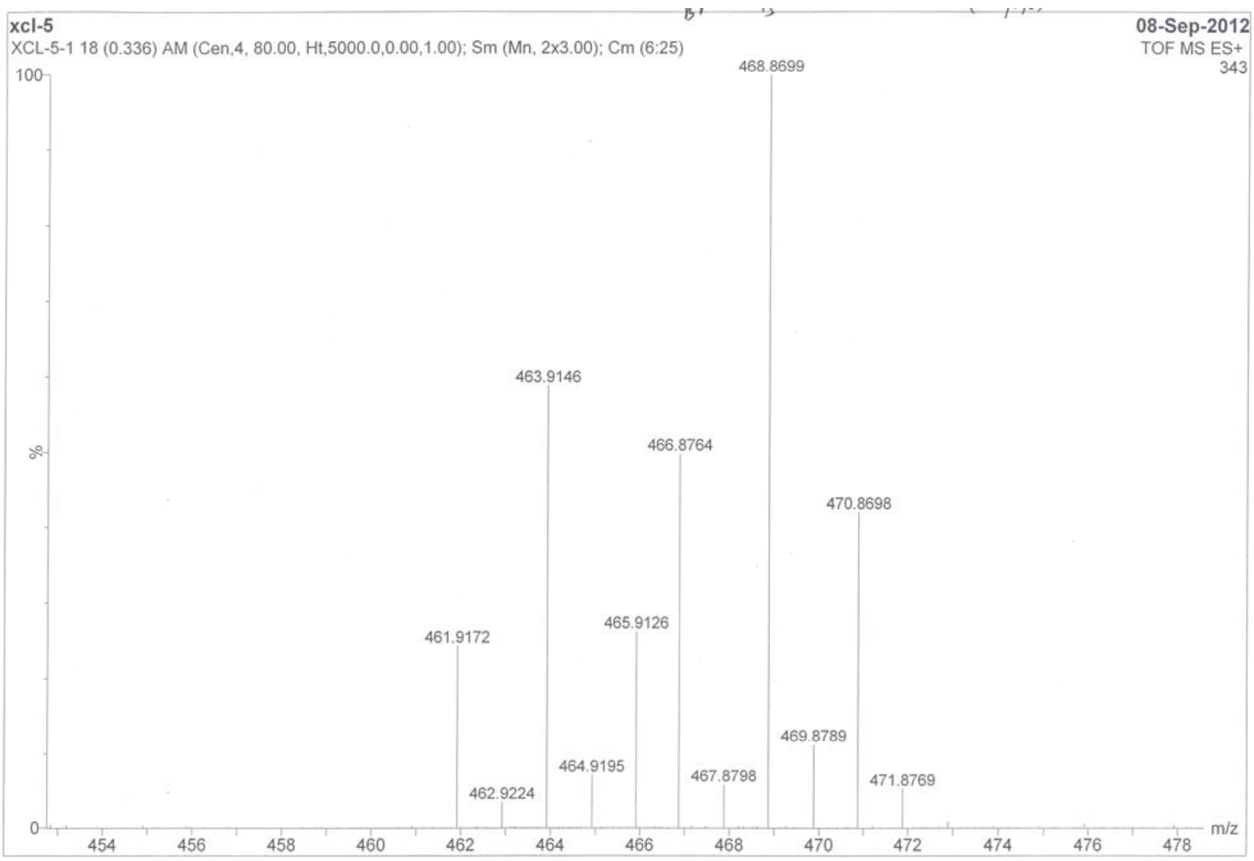

HRMS of compounds **3f**

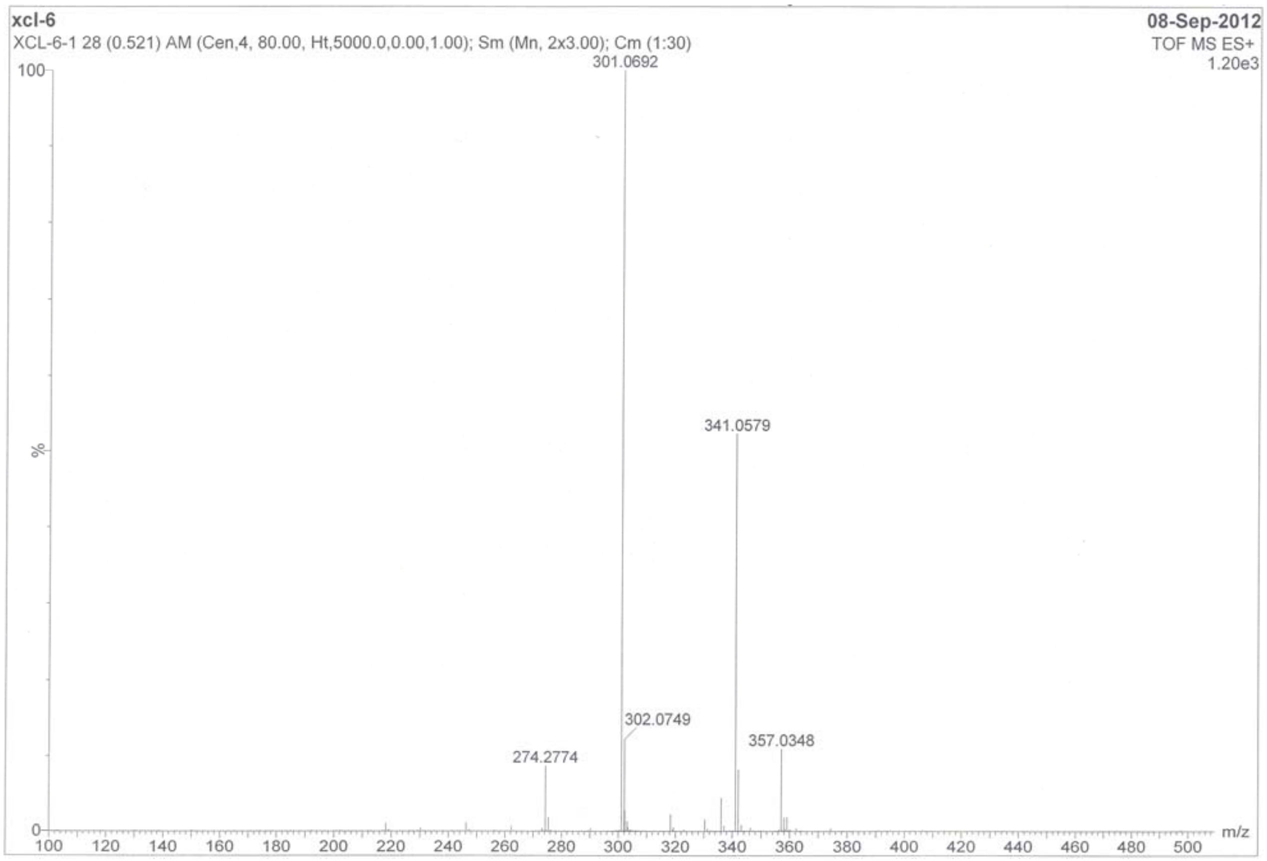

HRMS of compounds **3g**

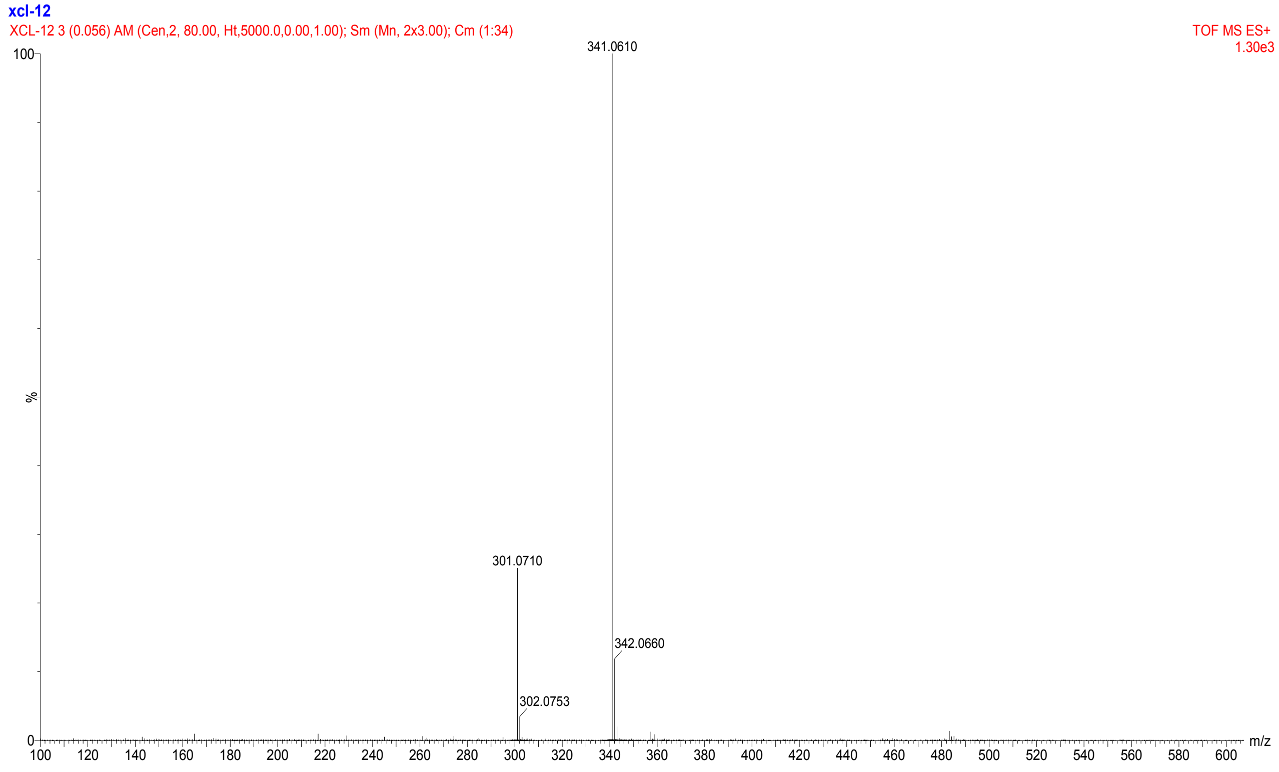

HRMS of compounds **3h**

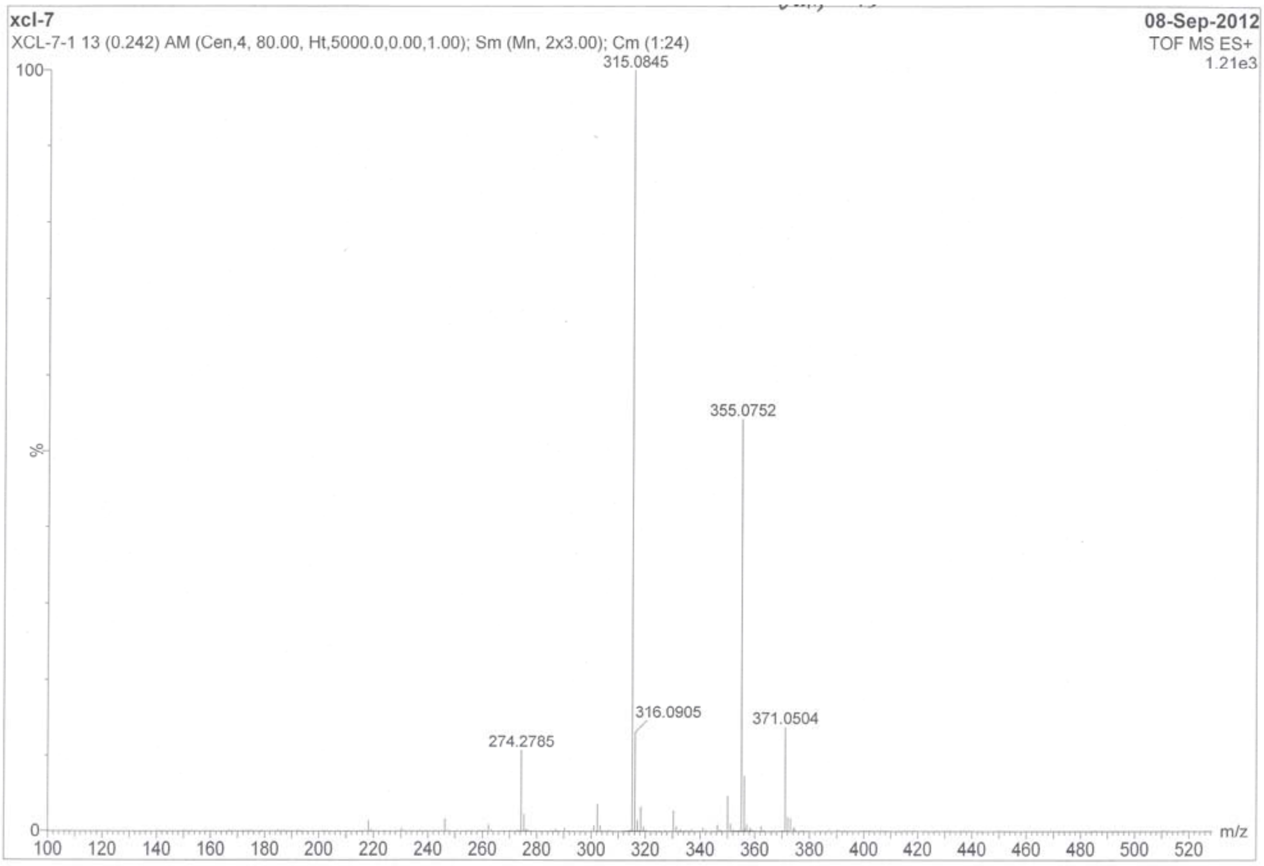

HRMS of compounds **3i**

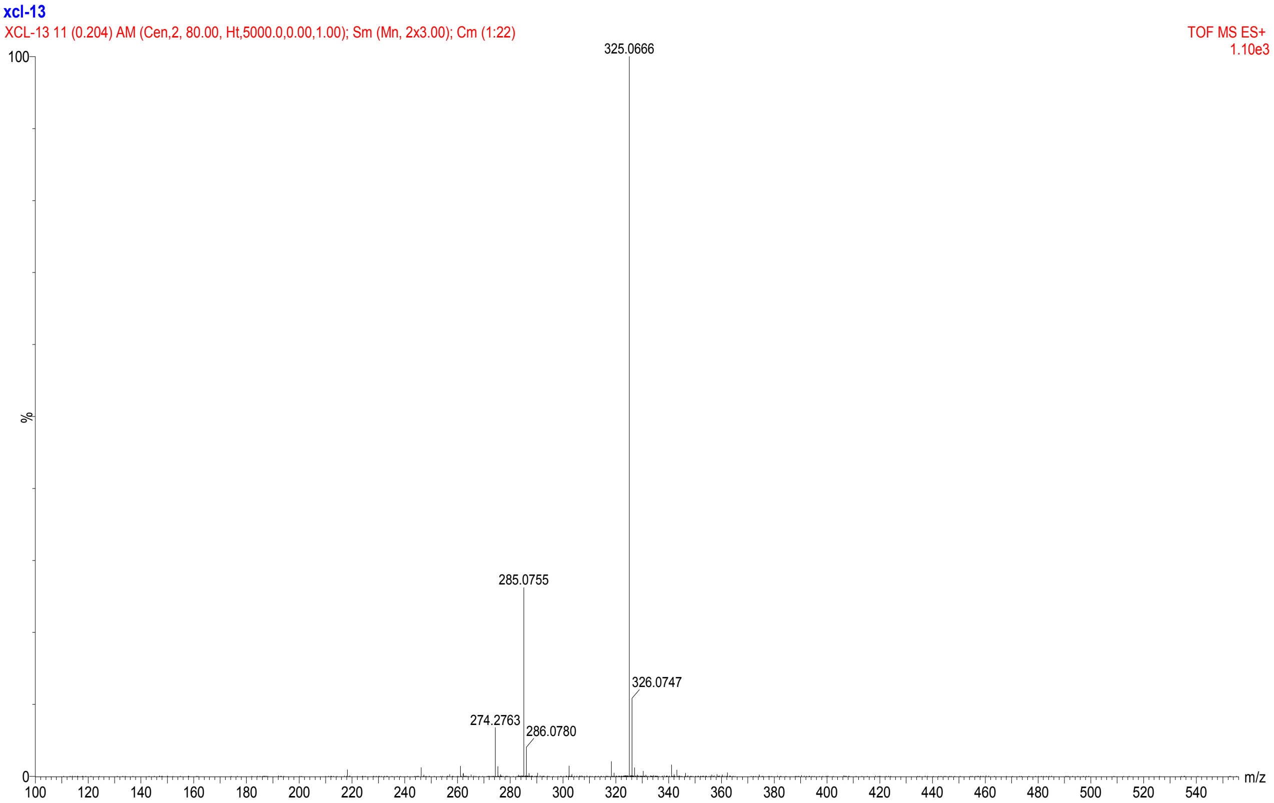

HRMS of compounds **3j**

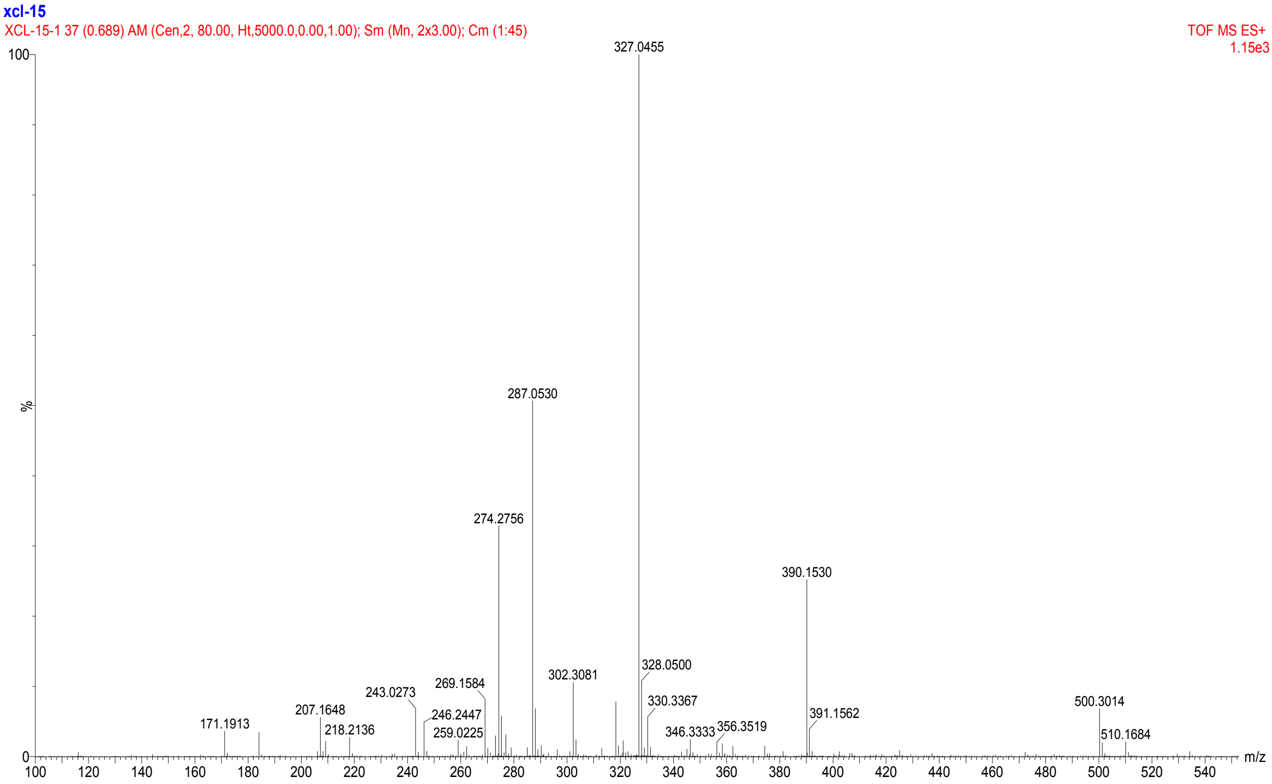

HRMS of compounds **3k**

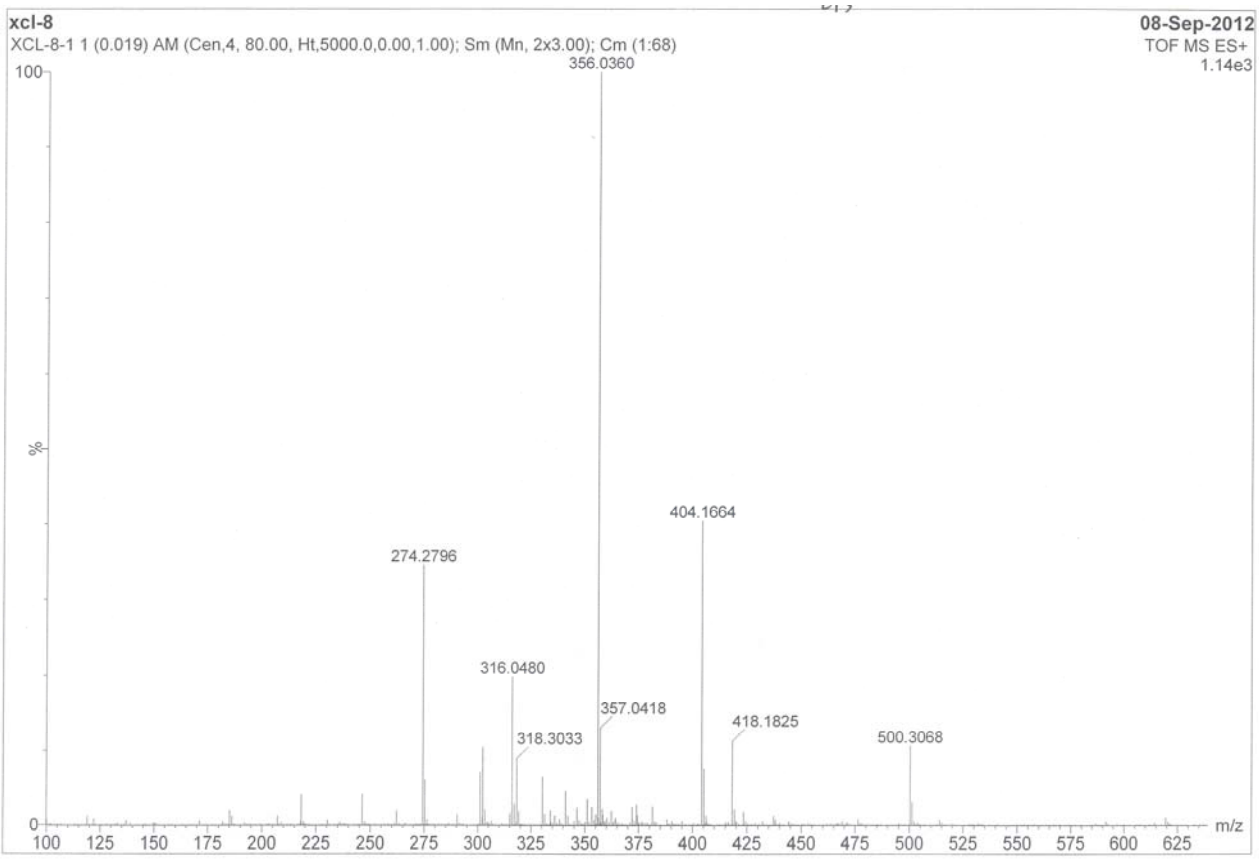

HRMS of compounds **3l**

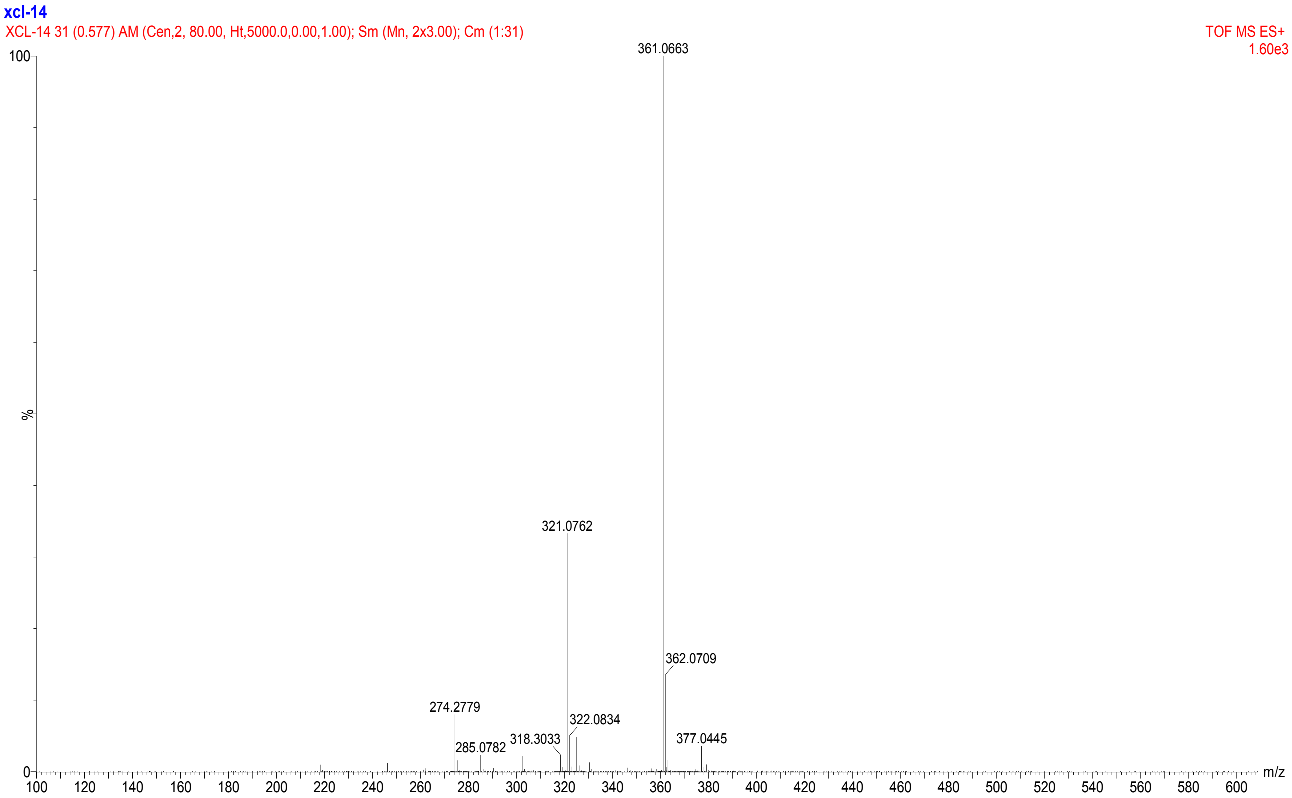

Supplement: Supplementary file 1 [file molecules-18-11964-s001.pdf]
